# CAR-modified marrow infiltrating lymphocytes efficiently target malignant plasma cells with very low antigen density

**DOI:** 10.1016/j.ymthe.2026.02.004

**Published:** 2026-02-06

**Authors:** Nicolas Camviel, Davide Gambarotto, Violaine Andre, Raphael Stadelmann, Christian Sieben, Raphael Gottardo, Suliana Manley, Caroline Arber

**Affiliations:** 1 Department of Oncology UNIL-CHUV, University Hospital Lausanne (CHUV) and University of Lausanne (UNIL), 1011 Lausanne, Switzerland; 2 Ludwig Institute for Cancer Research Lausanne, 1011 Lausanne, Switzerland; 3 Swiss Cancer Center Léman, 1011 Lausanne, Switzerland; 4 AGORA Cancer Research Center, 1011 Lausanne, Switzerland; 5 Institute of Physics, Ecole Polytechnique Fédérale Lausanne, 1024 Ecublens, Switzerland; 6 Service of Hematology and Central Laboratory of Hematology, Departments of Oncology UNIL-CHUV and Laboratory Medicine and Pathology, 1011 Lausanne, Switzerland; 7 Nanoscale Infection Biology Group, Helmholtz Centre for Infection Research, 38124 Braunschweig, Germany; 8 University Hospital Lausanne (CHUV), 1011 Lausanne, Switzerland; 9 University of Lausanne (UNIL), 1011 Lausanne, Switzerland; 10 Service of Immuno-Oncology, Department of Oncology UNIL-CHUV, University Hospital Lausanne, 1011 Lausanne, Switzerland

**Keywords:** chimeric antigen receptor, CAR, multiple myeloma, marrow infiltrating lymphocytes, super-resolution microscopy, antigen density, antigen sensitivity, CAR-T, APRIL-CAR

## Abstract

B cell maturation antigen (BCMA) is the primary target for chimeric antigen receptor (CAR)-T cell therapies and other immunotherapies in multiple myeloma (MM). Frequent relapses and the need for sequential treatments underscore the importance of alternative targets. We previously developed a dual-antigen-specific CAR recognizing both BCMA and transmembrane activator and CAML interactor (TACI). Here, we efficiently expressed this CAR in patient-derived marrow-infiltrating lymphocytes (MILs) to investigate MM cell killing via both endogenous T cell receptor (TCR) specificities and the CAR. While the patients’ MIL TCRs did not recognize or kill autologous malignant plasma cells (PCs), the CAR consistently mediated efficient PC killing even at low or undetectable antigen levels by flow cytometry. Quantification of BCMA and TACI molecules on target cells by direct stochastic optical reconstruction microscopy (*d*STORM) revealed that both BCMA and TACI were significantly underestimated by flow cytometry. Analysis by *d*STORM allowed us to delineate the dual-antigen sensitivity of the CAR, indicating that CAR-MILs were polyfunctional and killed targets at very low antigen densities. Our findings support the further exploration of TACI as target in MM and underline the value of super-resolution microscopy in assessing antigen density thresholds for CAR-T cells.

## Graphical abstract

**Figure:**
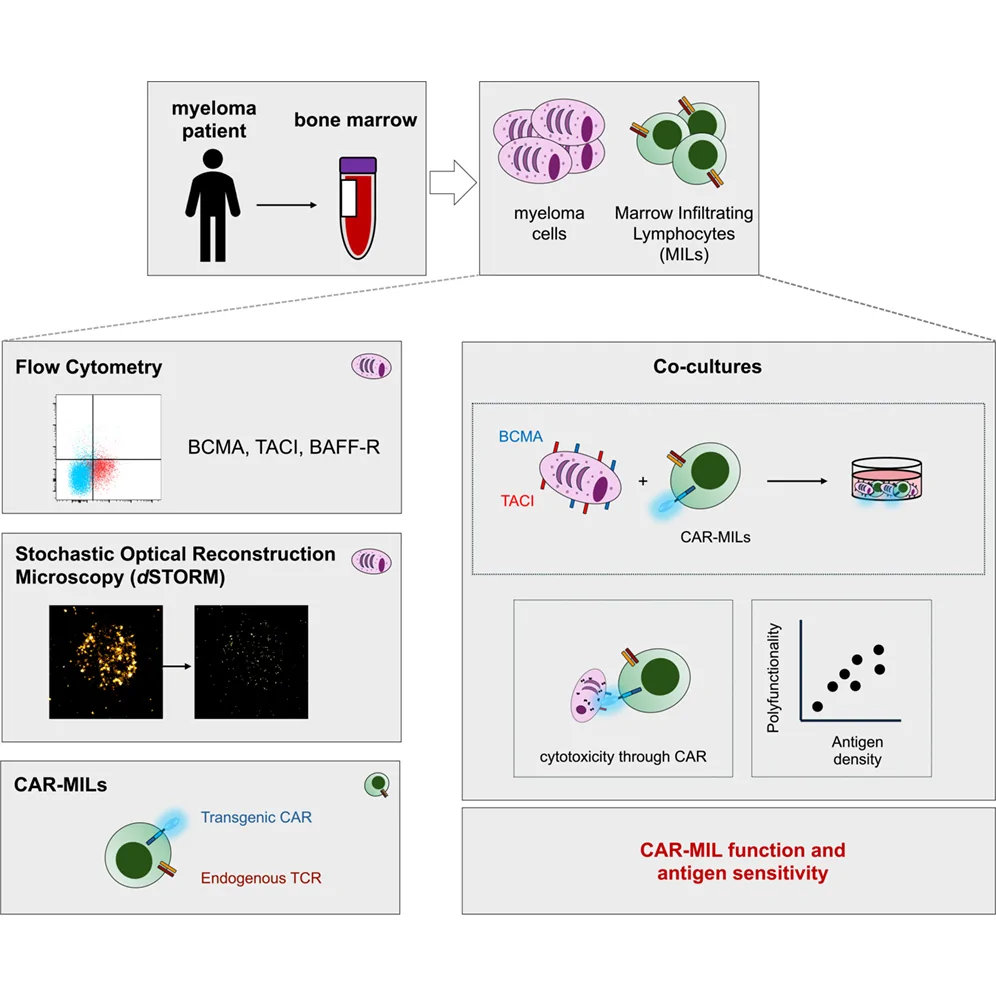

## Teaser

Multi-antigen targeted cellular therapies may tackle tumor heterogeneity. Harnessing the unique precision of super resolution microscopy, the antigen sensitivity of chimeric antigen receptor modified marrow infiltrating lymphocytes (CAR-MILs) of multiple myeloma (MM) patients was delineated. CAR-MILs were polyfunctional and killed primary autologous MM cells with very low target antigen densities.

## Introduction

Chimeric antigen receptor (CAR)-T cell therapies targeting B cell maturation antigen (BCMA) have changed the standard of care for patients with multiple myeloma (MM).^1^^,^^2^ While fast and deep responses can be achieved, disease relapse is often driven by mechanisms that include chronic antigen stimulation and CAR-T cell exhaustion, BCMA downmodulation, or genetic loss.^3^ Another hurdle remains the BCMA target antigen heterogeneity among patients’ malignant plasma cells (PCs), likely also impacting CAR-T cell therapy outcomes.^3^ A potential solution to overcome these problems are multi-antigen targeted T cell therapies that tackle several antigens simultaneously. Among others, receptors from the tumor necrosis factor (TNF) receptor family, including BCMA, transmembrane activator and CAML interactor (TACI), and B cell activating factor receptor (BAFF)-R have been explored as combinatorial CAR-T cell targets in MM.^4^^,^^5^^,^^6^^,^^7^^,^^8^^,^^9^^,^^10^

To exploit endogenous T cell receptor (TCR) specificities, autologous bone marrow (BM) could be used as the source of marrow-infiltrating lymphocytes (MILs).^11^ In analogy to tumor-infiltrating lymphocytes in solid tumors, MILs in hematologic malignancies are thought to contain T cells with αβ-TCR specificities recognizing epitopes from tumor-derived specific neoantigens or autologous tumor-associated antigens.^12^^,^^13^^,^^14^ Expanded MILs were cytotoxic against autologous patient-derived malignant PCs and were mostly of central memory (CM) phenotype. MILs could be exploited for adoptive T cell therapy in MM as a previous clinical trial has shown intriguing results with *ex vivo* expanded MILs in the context of autologous hematopoietic stem cell transplantation.^15^ Since little is known about gene-modified MILs, we hypothesized that (1) MILs offer a patient-derived autologous T cell source for CAR modification, enabling multi-antigen targeting of MM, and that (2) direct stochastic optical reconstruction microscopy (*d*STORM) analysis of primary patient samples more reliably determines CAR-MIL target antigen sensitivity than flow cytometry (FCM).^6^

To address our hypotheses, we (1) explored the feasibility of CAR-MIL generation from patient BM, (2) assessed CAR-MIL cytotoxicity and whether CAR-MILs kill autologous malignant PCs through the CAR and/or the endogenous TCRs, and (3) investigated CAR target antigen densities on patient samples by both FCM and *d*STORM and corroborated findings with CAR-MIL polyfunctionality and cytotoxicity.

We found that MILs were very efficiently transduced and expanded *in vitro* with our previously characterized dual-antigen-specific APRIL (a proliferation-inducing ligand)-CARs^6^ and that CAR-MILs were cytotoxic against autologous malignant PCs. In addition, APRIL-CAR MILs were able to recognize and kill PCs with very low antigen densities as detected by *d*STORM. Cytotoxicity was mediated through the CAR, not through the MIL TCRs. Our findings demonstrate that super-resolution microscopy performed on patient samples can give unprecedented insights into BCMA and TACI antigen densities and can be useful to delineate antigen sensitivity of novel CARs undergoing preclinical evaluation.

## Results

### BCMA, TACI, and BAFF-R expression on primary malignant PCs was heterogeneous

**Figure 1:**
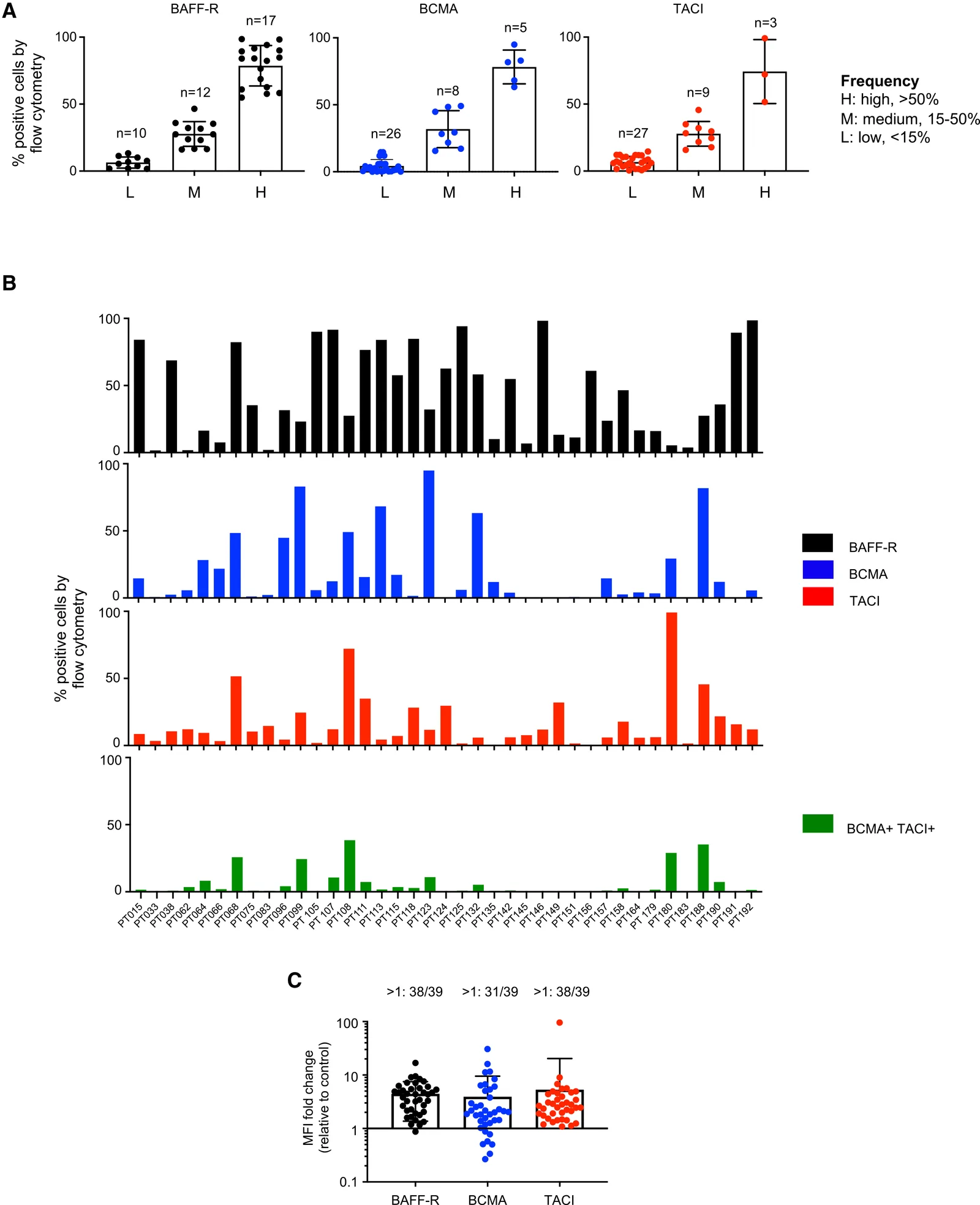
Heterogeneous expression of BAFF-R, BCMA, and TACI on malignant plasma cells (PCs) of myeloma patients by flow cytometry (A) Bar charts depict percent positive cells for BAFF-R, BCMA, and TACI, gated on the CD138^+^CD38^+^ malignant PC population in MM patient bone marrow (BM). Samples were classified into subgroups according to the frequency of marker positive cells: low (<15% positive cells), medium (15%–50% positive cells), or high (>50% positive cells). Symbols represent individual values, and bars show mean ± SD. (B) For each patient, expression of BAFF-R (black), BCMA (blue), TACI (red), or BCMA and TACI double positivity (green) is shown as percent positive cells of the CD138^+^CD38^+^ population. (C) Expression level of BAFF-R, BCMA, and TACI based on mean fluorescence intensity (MFI) fold change relative to control. *n* = 34 MM patients at diagnosis; *n* = 5 at first relapse.

BCMA, TACI, and BAFF-R belong to the TNF receptor family and are critically involved in B cell maturation, survival, and PC maintenance. These B cell lineage-restricted receptors are heavily investigated targets for CAR-T cell therapy of MM, B cell malignancies, and autoimmune diseases. We therefore scrutinized BM aspirates of MM patients at diagnosis (*n* = 34) or at first relapse (*n* = 5) for BCMA, TACI, and BAFF-R expression on CD138^+^CD38^+^ malignant PCs by FCM (gating strategy and individual examples in Figure S1) and classified the frequency of marker-positive cells in low (L, <15% positive cells), medium (M, 15%–50% positive cells) or high (H, >50% positive cells). BAFF-R^+^ populations were more frequently detected than BCMA^+^ or TACI^+^ populations, with 17/39 patients (43.5%) having a high frequency of BAFF-R^+^ populations, compared with only 6/39 patients (15.4%) and 3/39 patients (7.7%) with a high frequency of BCMA^+^ and TACI^+^ populations, respectively (Figure 1A). In some patients, we detected all three receptors on PCs, in others only one, two, or none (Figure 1B). However, when analyzing expression levels based on mean fluorescence intensity (MFI), we found that BAFF-R, BCMA, and TACI are expressed in almost all samples, although at variable intensities (above 1-fold change compared with the CD3^+^ T cell control population: BAFF-R: 38/39, 97.4%; BCMA: 31/39, 79.5%; TACI: 38/39, 97.4%). BAFF-R was the antigen with the highest fold change level of expression on PCs (at least 5-fold change in expression compared with the CD3^+^ control population: BAFF-R, 13/39, 33.3%; BCMA, 10/39, 25.6%; TACI, 5/39, 12.8%) (Figure 1C). These results suggest that BAFF-R is an interesting alternative target for immunotherapies of MM.

### CAR-MILs were efficiently generated from patient BM aspirates

**Figure 2:**
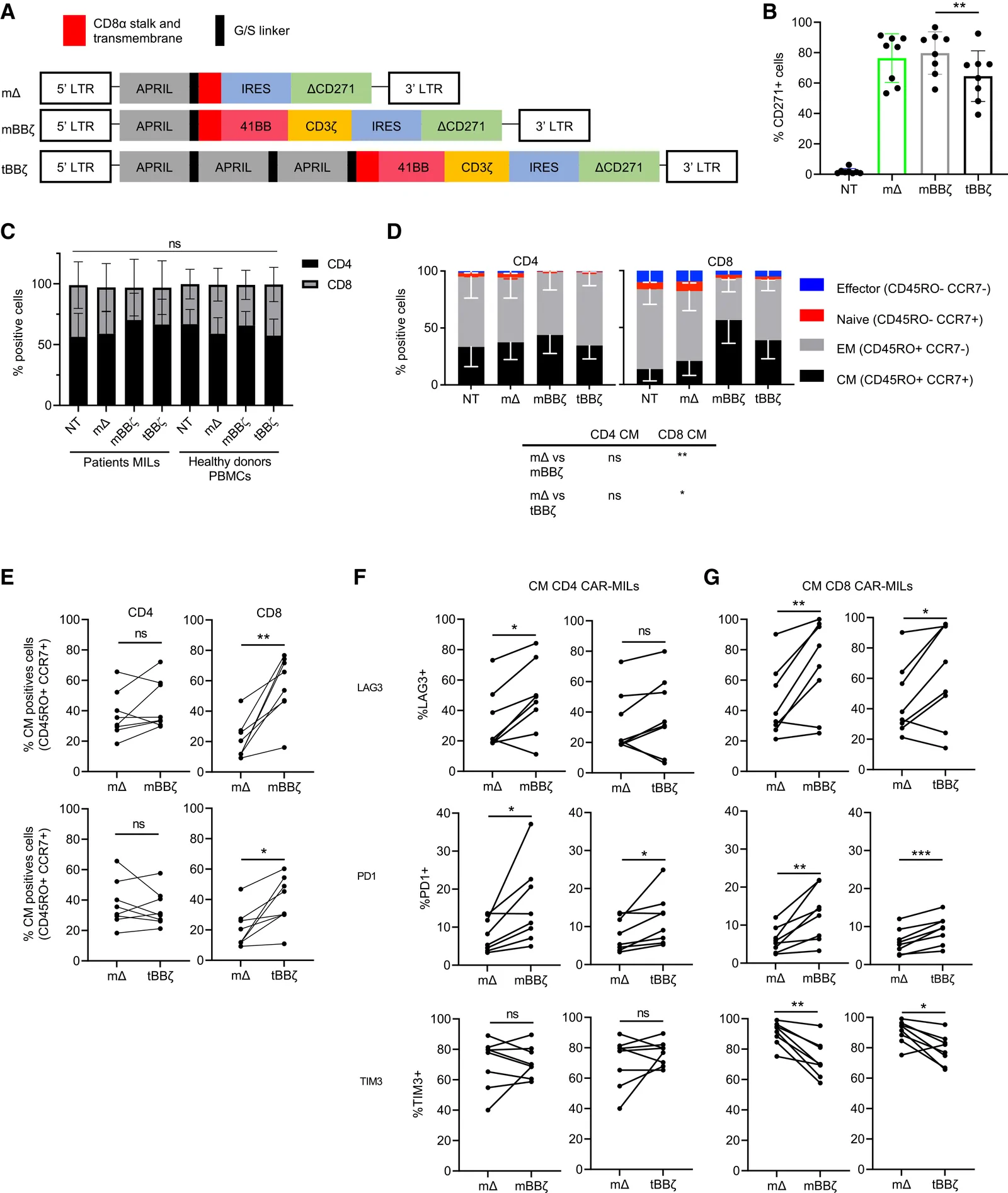
Generation and phenotypic characterization of APRIL CAR-MILs (A) Scheme of CAR constructs. Control non-signaling mΔ APRIL CAR and two 41BB-ζ-based APRIL CARs, in monomeric (m) or trimeric (t) configuration. (B) CAR transduction efficiency in activated MILs by CD271 staining. (C) CD4/CD8 distribution in CAR-MILs or CAR-T cells derived from healthy donor PBMCs. (D and E) (D) Stacked bars or (E) line graphs analyzing differentiation phenotype in CD4 or CD8 CAR-MILs: effector (CD45RO^−^CCR7^−^), naive (CD45RO^−^CCR7^+^), effector memory (EM; CD45RO^+^CCR7^−^), and central memory (CM; CD45RO^+^CCR7^+^). (F and G) Line graphs show comparison of exhaustion markers (LAG3, PD1, and TIM3) in CM (CCR7^+^CD45RO^+^) population of CD4 (F) or CD8 (G) CAR-MILs. (B–G) *n* = 8 MM patients, paired *t* test. Bars show mean ± SD. *n* = 6 healthy donors. Paired *t* test, significance level: ns, not significant, ∗*p* < 0.05, ∗∗*p* < 0.01, and ∗∗∗*p* < 0.001.

We next aimed to generate multi-antigen-specific T cells by leveraging both endogenous and engineered specificities through TCR and CAR, respectively. PC depleted CD138^−^ BM fractions from selected MM patients were polyclonally activated and retrovirally transduced with our previously developed APRIL-CARs^6^ in different formats: mBBζ (monomeric [m] APRIL as ligand binding domain), tBBζ (trimeric [t] APRIL as a ligand binding domain), or mΔ non-signaling control CAR (Figure 2A). We obtained high transduction efficiencies with 76.4% ± 15.9% for mΔ, 79.7% ± 13.9% for mBBζ, and 64.5% ± 16.6% for tBBζ CARs (*n* = 8) (Figure 2B), with the tBBζ construct reaching significantly lower efficiencies than mBBζ, likely due to construct size. At the end of expansion, we obtained CAR-MILs with a predominant (>50%) CD4^+^ CAR^+^-T cell population, similar to results obtained with healthy donor peripheral blood mononuclear cells (PBMCs) (Figure 2C). In the CD8^+^ compartment, both mBBζ and tBBζ CARs drove significant enrichment in CM (CD45RO^+^ CCR7^+^) phenotype (Figures 2D [summary] and 2E [individual donors]), comparable to what we previously described in healthy donor PBMCs. The profiling of the activation/exhaustion markers LAG3, PD1, and TIM3 on CAR-MILs revealed that both PD1 and LAG3 were expressed on a significantly higher proportion of mBBζ than on control mΔ CAR-MILs (Figures 2F and 2G), while the proportion of TIM3^+^ mBBζ MILs was lower than controls in the CD8^+^ compartment. Thus, we efficiently generated CAR-MILs from BM aspirates enriched for CM phenotype T cells.

### Cytotoxic activity of CAR-MILs against autologous malignant PCs was mediated by the CAR

**Figure 3:**
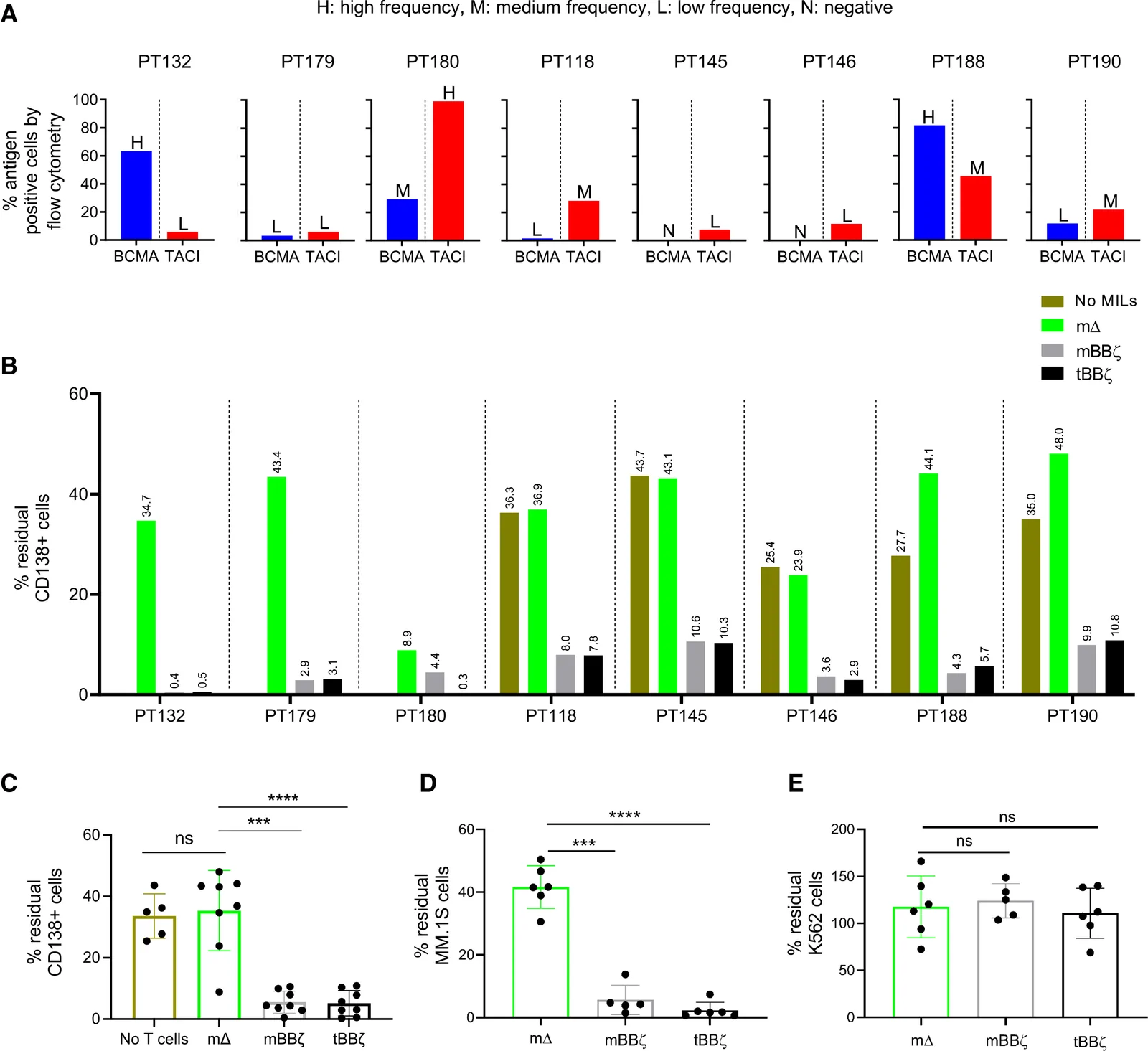
Primary malignant PCs with very low BCMA and/or TACI MFIs are efficiently eliminated by autologous CAR-MILs (A) Frequency of BCMA^+^ and TACI^+^ PCs by flow cytometry, and classification into high (H), medium (M), and low (L) frequency or negative (N). (B) Residual CD138^+^ PCs after 24 h of co-culture with CAR-MILs (mΔ: non-signaling control CAR, all samples) or no MILs as additional control (patients [PTs] 118, 145, 146, 188, and 190). Results from individual patient samples are shown. N/A, not applicable; the no-MILs condition was not assessed for patients 132, 179, and 180. (C) Summary bar graph of the data shown in (B). (A–C) *n* = 8 patients, individual values (A and B) and mean ± SD and individual data points as dots (C). (D and E) Residual MM.1S (D) and K562 (E) target cells after 24 h of co-culture with CAR-MILs from patients 118, 145, 146, 179, 188, and 190. (C–E) Paired *t* test, significance level: ns, not significant, ∗∗∗*p* < 0.001, and ∗∗∗∗*p* < 0.0001.

We next investigated the cytotoxicity of CAR-MILs against autologous tumor targets. Three patient samples with low, two with medium, and three with high frequencies of BCMA^+^ and/or TACI^+^ PCs by FCM were selected (Figure 3A). We found that CAR-MILs from all eight patients recognized and killed autologous tumor cells *in vitro* (Figures 3B [individual samples] and 3C [summary]), regardless of the frequency of the BCMA^+^ and/or TACI^+^ population by FCM (Figure 3A), indicating that target antigen density could play an important role in this *in vitro* killing assay. In five patients (nos. 118, 145, 146, 188, and 190), we also evaluated whether part of this cytotoxicity was mediated by the endogenous MIL TCRs. We found no difference in the remaining live tumor cells in co-cultures with no MILs, non-transduced MILs, or MILs transduced with non-signaling mΔ CAR, indicating no detectable killing in the absence of a signaling CAR (Figures 3B, 3C, and S2). Moreover, we performed sequencing of the TCR β chains of the non-transduced, the mΔ CAR, and the BBζ CAR MILs from three patients (nos. 118, 188, and 190), revealing high polyclonality and TCR diversity in all samples. We identified more than 1 × 10^5^ clonotypes in 8 of 9 samples (Figure S3A). No clone was enriched to more than 4.5% in any sample (Figure S3B). We also calculated the Shannon entropy index weighting the abundance of each CDR3 sequence in each sample. Shannon entropy values have been reported in the range of 8.1–18.7 for the general population.^16^ In our analysis, we found values ranging from 11.9 to 14.2, confirming the high diversity of the TCR repertoire in our samples (Figure S3C). We performed cytotoxicity assays with CAR-MILs as controls from six patients against BCMA^+^TACI^+^ MM.1S cells and the antigen-negative K562 cells. We found efficient killing of MM.1S but not of K562 cells with CAR-MILs (Figures 3D and 3E). In two patients, CAR-MILs were also generated with an irrelevant control CAR targeting c-type lectin 1 (CLL1) on myeloid cells (CLL1-BBζ; Figure S4A). Again, no killing of autologous malignant PCs was observed in co-cultures with CLL1-BBζ CAR-MILs (Figure S4B). Taking together all these findings, we conclude that the endogenous TCR in the CAR-MILs we analyzed did not contribute to target cell killing.

### BCMA and TACI levels were heterogeneous on primary malignant PCs as detected by *d*STORM

**Figure 4:**
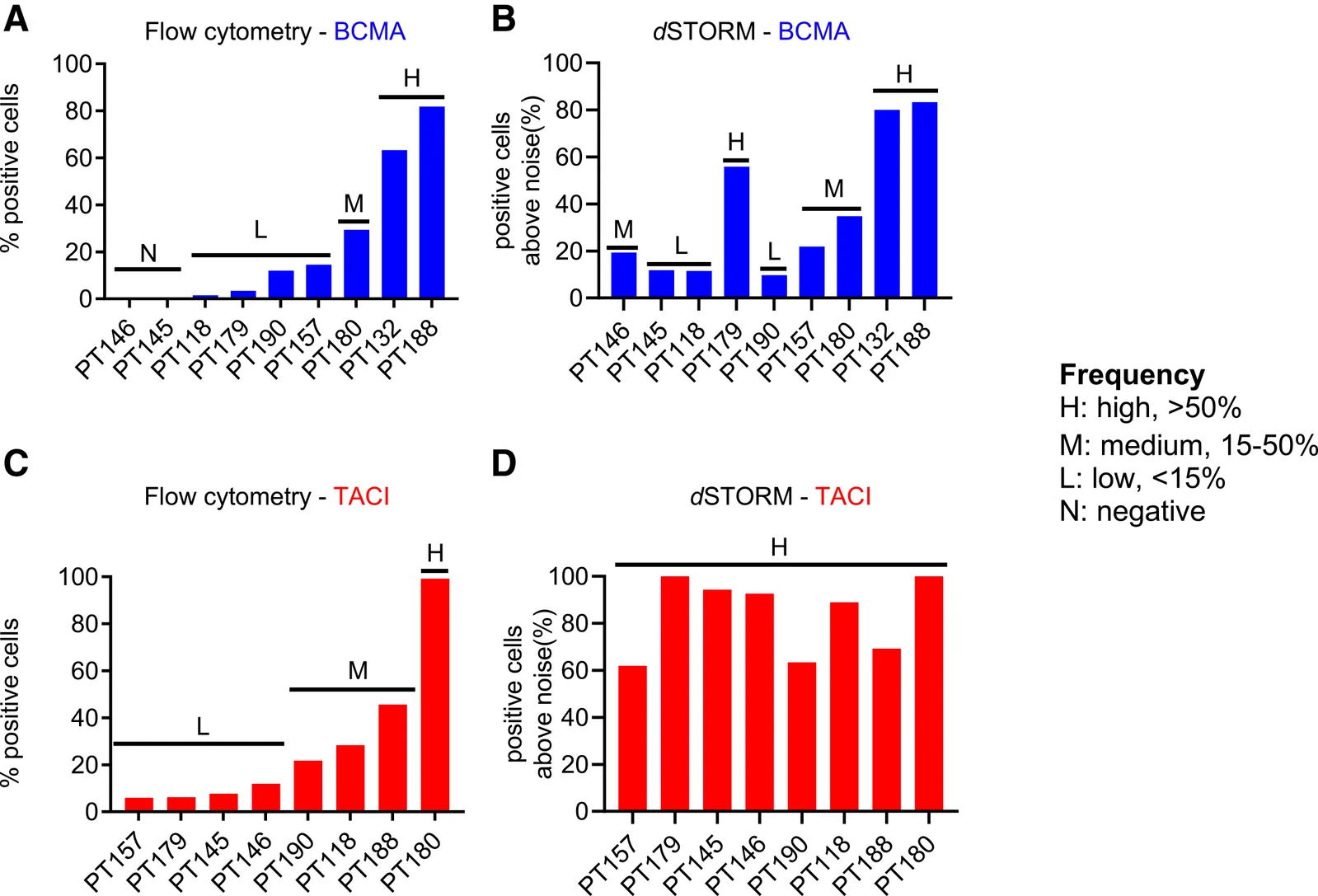
Flow cytometry underestimates the frequency of BCMA^+^ or TACI^+^ malignant PCs (A) Frequency of BCMA^+^ PCs in patient BM by flow cytometry (*n* = 9). (B) Frequency of BCMA^+^ PCs in patient BM by *d*STORM (*n* = 9). (C) Frequency of TACI^+^ PCs in patient BM by flow cytometry (*n* = 8). (D) Frequency of TACI^+^ PCs in patient BM by *d*STORM (*n* = 8).

The cytotoxicity experiments showed that CAR-MILs very efficiently killed primary malignant PCs *in vitro* even in patients with small BCMA and/or TACI populations by FCM. We hypothesized that BCMA and/or TACI antigen levels are likely below FCM detection limits. *d*STORM allows the accurate detection of single molecules of interest on the cell surface at a much higher sensitivity than FCM. For example, *d*STORM imaging of CD19 on MM samples previously unraveled ultra-low CD19 levels that were targetable with CD19-CARs.^17^ Thus, we turned to *d*STORM to measure both the frequency and antigen density of BCMA and TACI on the samples that were used in the cytotoxicity assays. Titration of anti-BCMA, anti-TACI, and matched isotype antibodies was performed on the MM.1S cell line expressing both BCMA and TACI (Figure S5A), and the staining protocol was applied to the patient samples. We first analyzed the frequency of BCMA^+^ or TACI^+^ cells by *d*STORM compared to the frequency by FCM (Figures 4A–4D). In most samples, the frequency of BCMA^+^ PCs was underestimated by FCM, and higher frequencies were measured by *d*STORM (Figures 4A and 4B). For TACI^+^ PCs, the difference between FCM and *d*STORM was even more striking, as by *d*STORM all samples analyzed contained more than 50% TACI^+^ PCs (Figures 4C and 4D).

**Figure 5:**
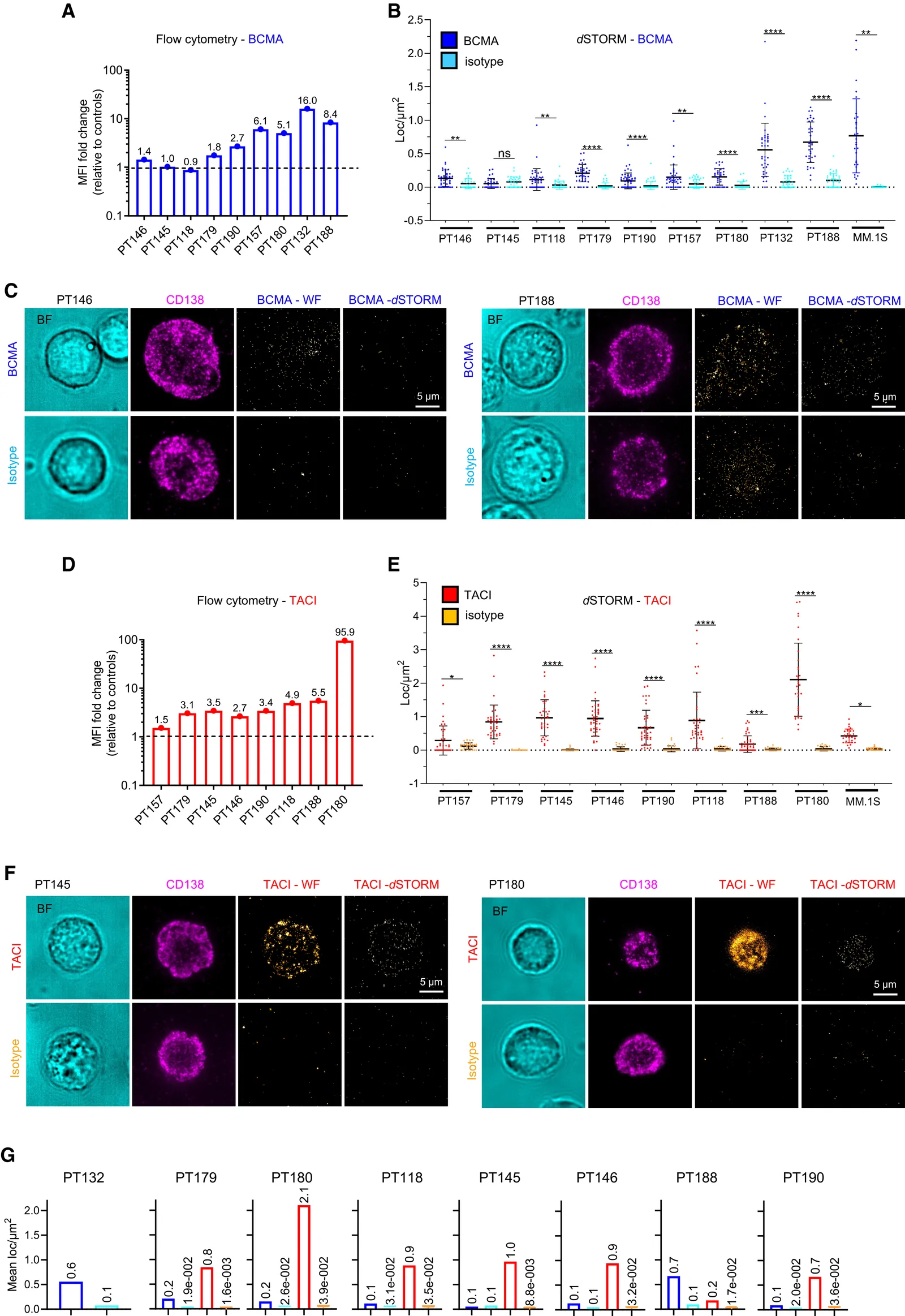
Flow cytometry underestimates the antigen density of BCMA or TACI on malignant PCs (A) BCMA MFI fold change relative to control by flow cytometry by sample. (B) BCMA antigen density by *d*STORM (loc/μm^2^), mean ± SD, and individual data points as dots for each PC or MM.1S cell analyzed. (C) Representative microscopy images for BCMA and isotype staining. From left to right: BF, bright-field image; CD138 staining to identify PCs; WF, wide-field image; and *d*STORM super-resolution reconstructed image for BCMA. (D) TACI MFI fold change relative to control by flow cytometry by sample. (E) TACI antigen density by *d*STORM (loc/μm^2^), mean ± SD, and individual data points as dots for each PC or MM.1S cell analyzed. (F) Representative microscopy images for TACI and isotype staining. From left to right: BF, bright-field image; CD138 staining to identify PCs; WF, wide-field image; and *d*STORM super-resolution reconstructed image for TACI. (G) Summary of the mean antigen densities for each patient sample used in the cytotoxicity assay with CAR-MILs. (B and E) Unpaired *t* test, significance level: ns, not significant, ∗*p* < 0.05, ∗∗*p* < 0.01, ∗∗∗*p* < 0.001, and ∗∗∗∗*p* < 0.0001.

Next, we analyzed antigen densities and compared *d*STORM signal localizations per μm^2^ (loc/μm^2^) to FCM MFIs. For BCMA, patients 145 and 118 did not have detectable BCMA FCM MFIs (Figure 5A), while by *d*STORM, only patient 145 did not have detectable BCMA over isotype staining (Figure 5B). The highest BCMA densities and MFIs were found in patients 132 and 188 by both FCM and *d*STORM; densities found on the MM.1S cell line are shown as a reference (Figures 5A and 5B). Of note, the BCMA MFI fold change by FCM was very low in patients 146 and 179 and undetectable in patient 118, whereas *d*STORM clearly detected BCMA molecules on those cells (Figure 5B). Only patient 145 was negative by both *d*STORM and FCM. Representative microscopy images for BCMA are shown in Figures 5C and S5B. For TACI, we noted again a large discrepancy between FCM and *d*STORM results (Figures 5D and 5E). By *d*STORM, TACI was consistently detected at high levels among all patients analyzed (8/8 patients; Figures 5D and 5E), indicating an important underestimation of TACI MFIs by FCM; densities found on the MM.1S cell line are shown as a reference. Representative microscopy images for TACI are shown in Figures 5F and S5B.

Finally, corroborating *d*STORM antigen densities with the cytotoxicity results, we found that CAR-MILs were able to recognize and kill primary human PCs with a combined mean BCMA and TACI antigen density as low as 0.7615 loc/μm^2^ (patient 190) (Figure 5G). For patient 132, TACI density could not be determined by *d*STORM for technical reasons. Polyfunctionality of CAR-MILs was assessed by intracellular cytokine staining (ICS) after stimulation with autologous malignant PCs or the cell lines MM.1S as positive and K562 as negative controls. While all patient CAR-MILs killed their autologous tumor cells, only a subset of patient CAR-MILs were able to mount a strong polyfunctional response (Figure S6). With the caveat of analyzing a small sample size, we detected a correlation between CAR-MIL polyfunctionality by ICS and target antigen density by *d*STORM using the COMPASS algorithm.^18^ Target cells with higher combined BCMA and TACI density triggered a higher frequency of polyfunctional CAR-MILs in both CD4 and CD8 compartments (Figure S7). CAR-MILs from six patients were also evaluated for their polyfunctionality upon challenge with MM.1S or K562 cells as controls (Figure S8), corroborating the results found upon stimulation with autologous PCs.

Thus, BCMA and TACI levels were heavily underestimated in the FCM analysis. Super-resolution microscopy, however, enabled us to delineate the dual-antigen sensitivity of the APRIL-CARs and revealed that very low antigen densities (below 1 loc/μm^2^) were recognized by this CAR, resulting in efficient target cell killing of primary patient samples.

## Discussion

We evaluated the potential for MILs from MM patients as an alternative T cell type for the generation of multi-antigen specific CAR-T cells and correlated functional data on cytotoxicity and polyfunctionality, with target antigen densities measured by super-resolution microscopy. While CAR-MIL generation was fast and efficient, their anti-tumor function was exclusively mediated through the CAR, but not through the endogenous TCRs. BCMA and TACI target antigen densities correlated with CAR-MIL polyfunctionality. We confirmed high levels of BAFF-R expression on malignant PCs and found that TACI levels are underestimated by standard FCM, thus indicating that both BAFF-R and TACI are interesting alternative target antigens to explore for the immunotherapy of MM.

Super-resolution microscopy is a highly specialized technology that is rarely used to quantify therapeutic target antigens in oncology but that has previously revealed a rationale for targeting CD19 expressed at ultra-low levels on a fraction of MM patients.^17^ Here, we quantified by super-resolution microscopy BCMA and TACI receptor densities on primary human malignant PCs, two antigens therapeutically targeted with CARs. We found that TACI cell surface expression was underestimated in all but one sample by standard FCM when compared with *d*STORM. Our findings underline the interest of further exploring TACI as a target antigen for immunotherapy and CAR-T cell therapy of MM. TACI was previously shown to have a good safety profile for CAR-T cell therapy and could serve for coping with BCMA loss.^7^ For BCMA, levels on most samples were also underestimated by flow compared with *d*STORM. Strikingly, one sample that was classified as negative by flow had detectable BCMA levels by *d*STORM, and two samples with very low levels by flow had clearly higher levels by *d*STORM. The third receptor in this TNF-R family that we analyzed, BAFF-R, was only assessed by FCM, since our CAR targeted only BCMA and TACI. BAFF-R was detected in the majority of patient samples analyzed. BAFF-R was previously reported to be highly expressed on malignant but not on normal PCs^19^^,^^20^ and described as a potential therapeutic target antigen in MM due to receptor expression and the disease-promoting function of the soluble ligand BAFF in the BM.^21^ BAFF-R is also expressed at high levels in most mature and immature B cell malignancies, as well as normal B cells, and BAFF signals are involved in driving autoimmune diseases. Applications of BAFF-R CAR-T cells could therefore be rather broad, potentially including all these conditions.^22^ Indeed, BAFF-R CAR-T cells have been developed in either conventional or adapter CAR formats, based on antibody fragments or the natural ligand, and clinical translation and evaluation in clinical trials is ongoing.^8^^,^^9^^,^^23^^,^^24^

By expressing our APRIL-CAR in MILs, we hypothesized that killing of MM by CAR-MILs is mediated through both the CAR recognizing BCMA and TACI on MM and the endogenous TCRs recognizing MM-specific or MM-associated antigens presented in the context of human leukocyte antigen on MM cells. However, we found that the cytotoxic activity was exclusively mediated through the CAR and not through the TCR in any of the patient samples investigated. Potential explanations for this finding could be that (1) our sample size was small, with eight patients evaluated, and we do not know whether these MILs contained neoantigen specific TCRs or not; (2) the patient samples were collected at diagnosis, where mutational load and TCR specificities can be lower than in the relapsed/refractory setting^13^; or (3) the neoantigen TCR population was too small within the CAR-MIL product to result in a measurable cytotoxic response upon polyclonal activation and expansion. It was previously reported that the frequency of expanded T cell clones with unique TCRs recognizing and killing malignant PCs was extremely low in BM samples at diagnosis.^25^ A recent study, however, reported that non-gene modified expanded MILs from BM samples of MM patients at diagnosis mediated cytotoxicity against autologous malignant PCs *in vitro*.^14^ When engineered with a CAR, we found in our experiments that the contribution of endogenous TCR specificities to CAR-MIL cytotoxicity was negligible. TCR sequencing corroborated these findings, we documented a highly polyclonal TCR repertoire in three patient samples evaluated.

Since we obtained matched polyfunctionality and cytotoxicity data of patient CAR-MILs against autologous malignant PCs, together with *d*STORM analysis on BCMA and TACI antigen densities on the target cells, we attempted to corroborate antigen density with polyfunctionality. Indeed, we found correlations in both the CD4 and CD8 CAR-MIL compartment between the frequency of a highly polyfunctional and rather cytolytic subset with simultaneous detection of four or five positive markers (CD107a^+^, granzyme B^+^, perforin^+^, TNF-α^+^, with or without interferon-γ^+^ [IFN-γ^+^]) and the combined BCMA and TACI antigen density measured by *d*STORM. Even though the numbers are small, the results suggest an antigen density-dependent activation of the CAR-MILs. Interestingly, CAR-MILs from all patients were able to kill their autologous target cells even with very low antigen densities on target cells. Our study demonstrates the added value of super-resolution microscopy in defining BCMA and TACI target antigen thresholds required for CAR-MIL activation. A complementary approach to the screening of patient samples could be a recently published synthetic system called CombiCells.^26^ This system allows the titration of cell surface antigen densities by using engineered cell lines expressing a spycatcher on their cell surface in combination with titrated amounts of recombinant protein labeled with a spytag. The spytag will covalently bind to the cell surface spycatcher and display the target antigen on the cell surface. Engineered T cells can be stimulated with these CombiCells and characterized functionally including phenotype, cytokine production, and cytotoxic capacity.^26^

Overall, generation and expansion of CAR-MILs was feasible and yielded high transduction efficiencies. However, we were unable to detect a contribution of endogenous TCRs to the CAR-MIL cytotoxicity against autologous malignant PCs. Our study provides insight into single-molecule detection of BCMA and TACI on primary malignant PCs from MM patients using super-resolution microscopy. Our correlative studies suggest that antigen density is linked to polyfunctionality and engagement of cytolytic functions of APRIL CAR-MILs. While such complementary studies are still technically demanding, of low throughput, and not easily applicable to patient screening, they can inform on CAR antigen sensitivity and optimal T cell activation thresholds during preclinical CAR development. We also reveal that both TACI and BAFF-R are interesting alternative targets that could be explored more deeply for CAR-T cell therapies in plasma and B cell malignancies as well as autoimmune diseases.

## Materials and methods

### Cell lines

MM.1S and HEK293T cells were purchased from the American Type Culture Collection. K562 (chronic myeloid leukemia) was purchased from the German Cell Culture Collection. MM.1S and K562 cells were maintained in RPMI 1640 (Gibco, catalog no. 61870010) supplemented with 10% fetal bovine serum (FBS; Biowest, catalog no. S1810-500, or Gibco, catalog no. 10270106), 1% penicillin-streptomycin (Gibco, catalog no. 15140122), and 1% l-glutamine (BioConcept, catalog no. 5-10K00-H). MM.1S and K562 cells were authenticated by Microsynth in January 2022.^6^

### MM patient BM samples

BM samples of routine BM aspirates performed at the hematology service of the Lausanne University Hospital were obtained from MM patients under a research protocol approved by the local ethics committee (CER-VD 2018-01951). Cell surface phenotyping of selected markers was performed by FCM, as described below. Malignant PCs were isolated from whole BM by CD138^+^ selection (EasySep, STEMCELL Technologies, catalog no. 17877). The CD138^+^ fraction and the CD138^−^ fraction containing MILs and all other BM cells were aliquoted and frozen separately using 40% RPMI 1640 (Gibco, catalog no. 61870010), 50% FBS (Gibco, catalog no. 10270-106, lot no. 2166446), and 10% DMSO (Sigma-Aldrich, catalog no. 41639). Cells were stored in the vapor phase of liquid nitrogen until use. The CD138^−^ fraction was thawed and used to generate CAR-MILs, as described below. The CD138^+^ fraction containing malignant PCs was used for *d*STORM microscopy, for the ICS assay, or in co-culture experiments.

### FCM

Cell surface staining was performed on whole BM with specific antibodies for 15 min at 4°C with antibodies (Table S1). Other reagents used for FCM are listed in Table S1. Cell viability was determined using DAPI (BioLegend, catalog no. 422801) or Zombie UV (BioLegend, catalog no. 423107). The gating strategy to determine BCMA, TACI, and BAFF-R positivity included the CD3^+^ T cell population within the same sample as the negative control (Figure S1).

ICS was performed to assess CAR-MIL polyfunctionality. Protein trafficking was inhibited with GolgiPlug (BD, catalog no. 555020, diluted 10× in phosphate-buffered saline [PBS], 5 μL/mL) and GolgiDtop (BD, catalog no. 554724, diluted 10× in PBS, 5 μL/mL), and CAR-MILs from patients were stimulated with autologous CD138^+^ cells. The anti-CD107a antibody (BD, clone H4A3, catalog no. 563078) was added in the beginning of the co-culture (10 μL/mL), and the surface staining with CD3-Alexa Fluor 700 (BioLegend, clone 17A2, catalog no. 100216), CD4-phycoerythrin (PE)-Cy7 (BioLegend, clone OKT4, catalog no. 317414), CD8-BV605 (BioLegend, clone RPA-T8, catalog no. 301040), CD271-BV786 (BD, clone C40-1457, catalog no. 743361), and Zombie UV (BioLegend, catalog no. 423107) was performed after 6 h. Next, cell membranes were permeabilized with Cytofix/Cytoperm buffer (BD, catalog no. 554714) for 20 min at 4°C in the dark, after which samples were washed in 0.1% saponin (Sigma, catalog no. S7900-25G) containing FCM buffer (PBS with 2% FBS). Then, the intracellular staining was performed by adding anti-IFN-γ-BV711 (BD, clone 4S.B3, catalog no. 564793), anti-TNF-α-PE (BD, clone 6401.1111, catalog no. 340512), anti-perforin-Alexa Fluor 647 (BD, clone δG9, catalog no. 563576), and anti-granzyme B-fluorescein isothiocyanate (BD, clone GB11, catalog no. 560211) antibodies for 30 min at 4°C in the dark. Samples were washed twice with 0.1% saponin containing FCM buffer and resuspended in FCM buffer for analysis. Because of the limited material available, we could not perform the intracellular cytokine assay with the sample from patient 157.

Samples were acquired on a BD FCM LSR II, SORP, or Fortessa, using DIVA software (version 8.0.1). Data analysis was performed with FlowJo software (version 10 or higher). For graphical representation in pie graphs, ICS data were converted with Pestle software (version 2.0), and graphs were generated in SPICE software (version 6.0).

### Production of retroviral supernatant

Retroviral supernatants for APRIL-CARs were produced by transient transfection of 293T cells as previously described.^6^ Briefly, 293T cells reaching 50% confluency were co-transfected with (1) the RDF plasmid encoding the RD114 envelope protein, (2) the Peg-Pam plasmid encoding MoMLV gag-pol proteins, and (3) the SFG retroviral plasmid of interest (with long terminal repeat promoters and packaging signals), using GeneJuice transfection reagent (Merck, catalog no. 70967-3), according to the manufacturer’s instructions. After 48 and 72 h of culture, retroviral supernatants were harvested, filtered with a 0.45-μM filter (Filtropur S, Sarstedt, catalog no. 83.1826), snap-frozen on dry ice mixed with 100% ethanol, and stored at −80°C until use or used as fresh after filtration.

### Activation and retroviral transduction of MILs

CD138^−^ negative BM fractions were thawed and cells counted. Recovered cells were plated for T cell activation on non-tissue culture-treated plates coated with anti-CD3 (1 mg/mL, BioLegend, catalog no. 317347, clone OKT3) and anti-CD28 (1 mg/mL, BioLegend, catalog no. 302934, clone CD28.2) antibodies in T cell media (RPMI with 10% FBS, 2 mM l-glutamine, and 1% penicillin-streptomycin) supplemented with interleukin-15 (IL-15) and IL-7 (Miltenyi Biotec, 10 ng/mL each, catalog nos. 130-095-362 and 130-095-765, respectively) for 3 days. Twenty-four hours before transduction, retronectin (Takara Bio, catalog no. T100B) in PBS (7 μg/mL, 1 mL per well) was coated on a non-tissue culture-treated 24-well plate (Greiner Bio-One, catalog no. 662102) and incubated overnight at 4°C. For transduction, retronectin was removed, retroviral supernatant was added to the plate, and then was centrifuged for 1 h at 32°C. After careful removal of the retroviral supernatant, the suspension of activated MILs was added at 0.15 × 10^6^ cells/mL, and the plate was centrifuged for 10 min at 1,000 × *g* and 21°C. After 3 days at 37°C 5% CO_2_, transduced MILs were harvested and expanded in fresh T cell media containing IL-7 and IL-15 for 7 days before use in experiments.

### Co-culture killing assay

Primary MM cells derived from patients, or the cell lines MM.1S or K562, were co-cultured with autologous CAR-MILs at an effector-to-target ratio of 1:1 (depending on cell number available, 100,000, 50,000, or 20,000 cells each in a 96-well plate). After 24 h, residual primary MM cells and T cells were quantified by FCM using antibody staining and absolute counting beads (CountBright Beads, Life Technologies, catalog no. C36950).

### Correlation analysis between target antigen density and CAR-MIL polyfunctionality

First, for each cytokine combination and construct, the frequencies were calculated as the proportion of cells expressing the specific cytokine combination relative to the total CD4 (or CD8) MIL population. Second, the frequencies were background adjusted by subtracting the corresponding frequencies observed for the mΔ control construct. Spearman correlation coefficients and *p* values were calculated using the cor function in R. All plots and analysis were performed in R version 4.3.^18^

### Statistical analysis

Descriptive statistics were used for data analysis. For continuous variables, normality distribution was analyzed, and comparisons between two variables were made by *t* test. Analyses were performed in GraphPad Prism version 8.1.2 or higher. *p* values < 0.05 were considered significant, and the levels of significance are indicated in the figures and their legends.

### STORM

#### Preparation of labeled primary antibody

Unconjugated primary antibody raised against human BCMA (BioLegend, clone 19F2, 357502, mouse immunoglobulin G2a [IgG2a]), human TACI (BioLegend, clone 1A1, catalog no. 311902, rat IgG2a), and random mouse IgG2a (BioLegend, clone MOPC-173, catalog no. 400202) and rat IgG2a (BioLegend, clone RTK2758, catalog no. 400501) were directly labeled with Alexa Fluor 647 NHS ester (Invitrogen, catalog no. A37573). In the solution, 25 μg antibody were incubated for 1 h at room temperature with 50 mM borate buffer pH 8.5 (from DyLight Antibody Labeling Kits, Thermo Scientific, catalog no. J60803.AP) and 6 μg of the dye (for BCMA and isotype) or 4 μg of the dye (for TACI and isotype). Excess dye was removed with dye removal columns (Thermo Scientific, catalog no. 22858). The concentration of each labeled antibody was measured by ultraviolet-visible spectroscopy (Nanodrop2000, Thermo Fisher).

#### Antibodies and immunofluorescence staining protocol for MM.1S and primary MM patient cells

MM.1S and CD138^+^ selected patient cells were stained with Alexa Fluor 488 anti-human CD38 (BioLegend, clone HIT2, catalog no. 303512) at 2 μg/mL, PE anti-human CD138 (BioLegend, clone MI15, catalog no. 356504) at 0.5 μg/mL, and either Alexa Fluor 647 anti-human BCMA or Alexa Fluor 647 anti-human TACI. An antibody titration experiment was performed to obtain the minimum concentration of anti-BCMA and anti-TACI antibodies needed to label all molecules (Figure S5). MM.1S cells naturally expressing both BCMA and TACI receptors were stained with serial dilutions of Alexa Fluor 647 anti-human BCMA (0.1–5 μg/mL) or Alexa Fluor 647 anti-human TACI (0.5–15 μg/mL). We retained optimal antibody concentrations of 2.5 and 10 μg/mL for anti-human BCMA and anti-human TACI, respectively, for subsequent experiments. The same immunofluorescence protocol was used for both MM.1S and MM patient cells. Cells were resuspended at 1.5 × 10^6^ cells/mL before staining.

Patients’ cells were quickly thawed, diluted in excess of RPMI medium, centrifuged at 500 × *g* for 5 min, and re-diluted in fresh cold culturing medium (at 4°C) at the desired dilution. Cells were then deposited and stained on a 25-mm round coverslip (Marienfeld, no. 1.5H, catalog no. 0117650). Right before the experiment, coverslips were washed in absolute ethanol for 5 min in an ultrasonic cleaner, plasma cleaned, coated with 0.01% (w/v) poly-l-lysine (Sigma, catalog no. P8920) to maximize cell adhesion, and chilled at 4°C.

The immunostaining was performed as follows: antibodies were added to 100 μL cold medium with 1.5 × 10^5^ cells, and the whole volume was placed on a coated coverslip. Incubation proceeded for 3 h at 4°C to let the cells be stained and adhere on the coverslip. Next, cells were quickly and gently washed three times with 4°C cold PBS, fixed with pre-warmed (37°C) 4% paraformaldehyde (EMS, electron microscopy grade, catalog no. 15714) for 20 min at 37°C and washed three times in PBS. Coverslips were then stored in PBS at 4°C in sealed plates until they were imaged. Due to technical issues, patient 132 could not be screened for TACI density level.

#### *d*STORM imaging

Coverslips with stained cells were inserted in a custom-made support; incubated in the photo-switchable buffer based on 10 mM Tris in PBS, pH 7.5 containing 10 mM cysteamine, 50 mM β-mercaptoethanol, 2 mM cyclooctatetraene, 2.5 mM protocatechuic acid, and 125 nM protocatechuic dioxygenase, as previously described^27^; and sealed with parafilm to avoid oxygen exchange. The CD138-PE marker was used to find the focal plane where the cells contacted the coverslip, and a snapshot was taken. Then, the objective was moved by 600 nm to match the focal plane of the Alexa Fluor 647 channel, which is shifted due to chromatic aberration. The shift was determined using fluorescent beads and a cylindrical lens inserted into the detection path. Single-molecule localization microscopy imaging was performed using a custom-built flat-field epi-illumination microscope.^28^ A 642-nm laser (2RU-VFL-P-2000-642-B1R, MPB Communications) was used to switch off fluorophores within the sample, while a 405-nm laser (OBIS, Coherent) controlled the return rate of the fluorophores to the fluorescence-emitting state. A custom dichroic (ZT405/561/642/750/850rpc, Chroma) reflected the laser light and transmitted fluorescence emission before and after passing through the objective (CFI60 Plan Apo Lambda x60/numerical aperture 1.4, Nikon). After passing the emission filter (ET700/75M, Chroma), emitted light from the sample was imaged onto a scientific complementary metal oxide semiconductor (sCMOS) camera (Prime, Photometrics, pixel size 106 nm). For each field of view (74.2 × 74.2 μm), 40,000 frames at 10-ms exposure time were acquired, and at every 5,000 frames, the 405-nm laser was increased 0.5 mW. Images of the CD38 signal and the bright-field channel were captured at the middle plane of the cells.

#### Image reconstruction and data analysis

Image stacks were analyzed using sCMOS camera-specific single-molecule localization algorithms adapted from Huang et al.^29^ Next, localization density in CD38^+^CD138^+^ double-positive cells was analyzed as follows. Drift correction was obtained by redundant cross-correlation, the initial 1,000 frames were removed, and localizations with <500 photons per frame were discarded. Moreover, close repeated localizations were grouped using an alpha-shape algorithm (MATLAB), with an alpha value of 50, as described previously.^17^ Briefly, the alpha value was varied over a large range (0–1,000 nm), and the number of clusters per area was counted. At an alpha value of 50, the cluster density reached a maximum indicating successful grouping without merging clusters together. The localization density per cell was then measured by dividing the number of grouped localizations by the area of the cells contacting the coverslip, which was given by the CD138 fluorescence, manually cropped in ImageJ. Per each patient, between 30 and 60 cells were analyzed per staining (BCMA, TACI and isotype antibodies). To classify cells as positive for either BCMA, TACI, or isotype antibodies binding, the density of localizations inside cells was compared to the density of localizations in the area of the field of view (FOV) not covered by cells (considered to be noise). To do so, first, the localization density of the noise was measured in each FOV as (total FOV area − total area occupied by cells)/(total FOV localizations − localizations inside cells) = density noise. Second, for each FOV, using its density noise value, 100 simulations were run to determine whether the localization density observed in the cells may result from noise or a real signal. In each simulation, localizations were randomly positioned in the FOV area, maintaining the total density noise that was obtained experimentally, and the localization density in the cells was measured to extract the mean and the standard deviation (SD) of the 100 simulations. Since the probability that localizations from noise fall inside cells is uniform, simulated densities are Gaussian distributed. Thus, SD was used to produce a confidence interval, and cells were scored as positive when their localization density was higher than the mean density noise + two times the value of SD (sigma), which translates to a confidence interval of 97.5%.

### TCRβ sequencing and analysis

Bulk TCR sequencing analyses were performed using SEQTR, as described previously.^30^ Briefly, mRNA was isolated and amplified by *in vitro* transcription. The 5′ adapter was added by multiplex reverse transcription, and TCRs were amplified using one primer in the adapter and one in the constant region. Libraries were sequenced on an Illumina instrument and TCR sequences processed using an ad hoc Perl script. The Shannon entropy index was used to define the diversity of the repertoire as it considers both the number of TCR clonotypes *n* and the frequency *pᵢ* of each clone:

$$H=-\sum_{i=1}^{n}p_{i}\;\log\left(p_{i}\right)\text{,}$$

where *n* is the number of distinct categories (e.g., TCR clonotypes), and p_i_ is the probability or relative frequency of the *i* category.

## Data and code availability

All data will be made available upon reasonable request to the corresponding author.

## Acknowledgments

The authors would like to thank the medical team of the CHUV Hematology Service for their help with patient recruitment and obtaining patient informed consent. We also thank Juilette Griffie for advice with the imaging data analysis. We are very grateful for all the input and helpful discussions from the Arber lab members. We thank Christine Von Gunten and Lenka Polak for superb lab management. The project was in part supported by 10.13039/501100013362Swiss Cancer Research grant KFS-4542-08-2018-R and Blood Cancer United (formerly The Leukemia & Lymphoma Society) Translational Research Program grant no. 6676-24, both to C.A. In addition, C.A., R.S., and R.G. are supported by the 10.13039/501100006392Lausanne University Hospital and S.M. by the 10.13039/501100001703École Polytechnique Fédérale de Lausanne.

## Author contributions

N.C. and C.A. designed the study. N.C. performed experiments, D.G. performed microscopy, V.A. performed revision experiments, R.S. recruited and consented patients, C.S. was instrumental in setting up the *d*STORM imaging and data analysis pipeline, and R.G. performed correlative analysis of polyfunctionality and antigen density. N.C., D.G., V.A., R.G., S.M., and C.A. analyzed, interpreted, and discussed the results. N.C., D.G., and V.A. prepared the figures. S.M. and C.A. supervised the study. N.C., D.G., and C.A. wrote the manuscript. All authors edited the manuscript and agreed with the final version.

## Declaration of interests

C.A. has patents and patent applications in the field of engineered T cell therapies. C.A. receives licensing fees and royalties from Immatics (through her previous institution Baylor College of Medicine). C.A. has participated in advisory boards for Kite/Gilead, Janssen, and Celgene/BMS and received sponsored travel from Gilead (through her current institution University Hospital Lausanne). R.S. has participated in advisory boards for Incyte, AbbVie, Janssen, Takeda, BMS, and Stemline. None of these interests are related to the work presented in this paper.

## Supplemental information

Document S1. Table S1 and Figures S1–S8

Document S2. Article plus supplemental information
