# Supplementary material for: CAR-modified marrow infiltrating lymphocytes efficiently target malignant plasma cells with very low antigen density
Source: Mol Ther. 2026 Feb 6;34(6):3405–16. doi: 10.1016/j.ymthe.2026.02.004 (PMC13238939; doi:10.1016/j.ymthe.2026.02.004)
Supplement: Supplementary file 1 [file mmc1.pdf]

YMTHE, Volume *34*

Supplemental Information

CAR-modified marrow infiltrating lymphocytes efficiently target malignant plasma cells with very low antigen density

Nicolas Camviel, Davide Gambarotto, Violaine Andre, Raphael Stadelmann, Christian Sieben, Raphael Gottardo, Suliana Manley, and Caroline Arber

Table S1. List of antibodies and flow cytometry reagents.

Antibody	Company	Catalog number	Clone
CD3-BV711	Biolegend	#344838	SK7
CD4-PE-Cy7	Biolegend	#317414	OKT4
CD8-BV605	Biolegend	#301040	RPA-T8
CD271-PE	Biolegend	#345106	ME20.4
CD271-APC	Biolegend	#345108	ME20.4
CD271-BV786	BD	#743361	C40-1457
CD45RO-APC-Cy7	Biolegend	#304228	UCHL1
CD45RA-BV510	Biolegend	#304142	HI100
CCR7-FITC	BD	#561271	150503
CCR7-BV421	BD	#560863	150503
LAG3-FITC	Biolegend	#369308	11C3C65
PD-1-BV650	Biolegend	#329950	EH12.2H7
PD-1-Alexa Fluor 700	Biolegend	#329952	EH12.2H7
Tim-3-PE	Biolegend	#345006	F38-2E2
Tim-3-BV711	Biolegend	#345024	F38-2E2
CD138 PerCP Cy5.5	Biolegend	#356510	MI15
CD107a BV510	BD	#563078	H4A3
anti-TNF-α-PE	BD	#340512	6401.1111
anti-IFN-γ-BV711	BD	#564793	4S.B3
anti-Perforin-Alexa Fluor 647	BD	#563576	δG9
anti-GranzymeB-FITC	BD	#560211	GB11
CD267 (TACI) PE	BioLegend	#311906	1A1
CD267 (TACI) PE-Cy7	Biolegend	#311908	1A1
CD269(BCMA)-APC	BioLegend	#357506	19F2
CD269(BCMA)-PE	Biolegend	#357504	19F2
CD268 (BAFF-R)-FITC	BioLegend	#316904	11C1
CD38-BV711	BioLegend	#303528	HIT2
CD19-BV605	BioLegend	#302244	H1B19
CD3-AlexaFluor700	BioLegend	#300324	HIT3a
PE/Cyanine7 Rat IgG2aκ Isotype Ctrl antibody	Biolegend	#400522	RTK2758
PE Mouse IgG2aκ Isotype Ctrl (FC) antibody	Biolegend	#400214	MOPC-173
**Flow cytometry reagents**	**Company**	**Catalog number**	
CountBright Beads	Invitrogen	#C36950	
DAPI	Biolegend	#422801	
Zombie UV	Biolegend	#423107	
fixation buffer	BD	#554655	
Saponin	Sigma Life Sciences	S7900-25G	
Cytofix/Cytoperm™	BD	554714	

Figure S1 (1/3)

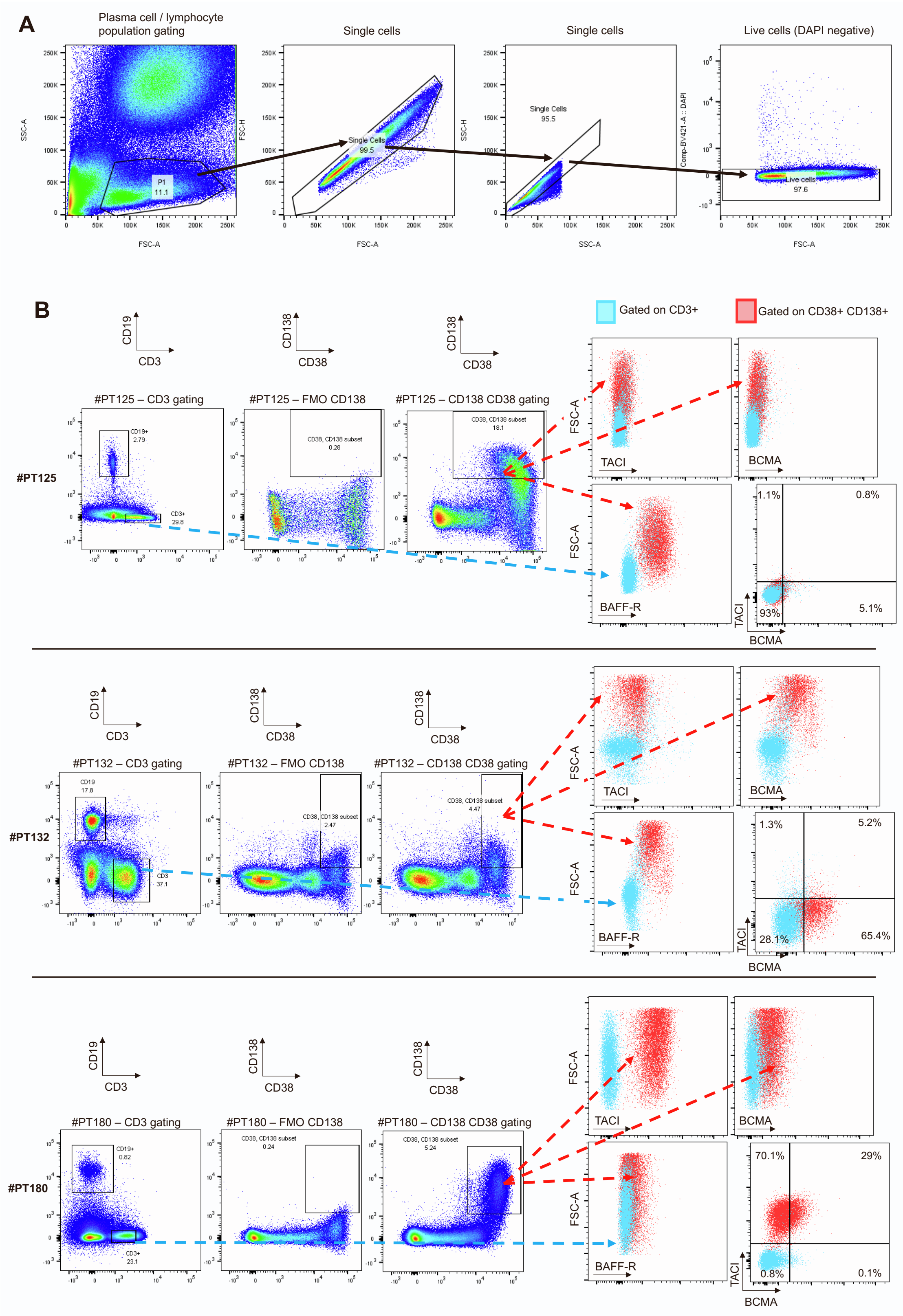

A
Plasma cell / lymphocyte population gating
Single cells
Single cells
Live cells (DAPI negative)
P1
11.1
Single Cells
99.5
Single Cells
95.5
Live cells
97.6
FSC-A
SSC-A
FSC-H
SSC-H
Comp-BV421-A :: DAPI
B
Gated on CD3+
Gated on CD38+ CD138+
CD19
CD3
CD138
CD38
#PT125 – CD3 gating
#PT125 – FMO CD138
#PT125 – CD138 CD38 gating
#PT125
CD19+
2.79
CD3+
29.8
CD38, CD138 subset
0.28
CD38, CD138 subset
18.1
FSC-A
TACI
BCMA
BAFF-R
1.1%
0.8%
93%
5.1%
#PT132 – CD3 gating
#PT132 – FMO CD138
#PT132 – CD138 CD38 gating
#PT132
CD19
17.8
CD3
37.1
CD38, CD138 subset
2.47
CD38, CD138 subset
4.47
1.3%
5.2%
28.1%
65.4%
#PT180 – CD3 gating
#PT180 – FMO CD138
#PT180 – CD138 CD38 gating
#PT180
CD19+
0.82
CD3+
23.1
CD38, CD138 subset
0.24
CD38, CD138 subset
5.24
70.1%
29%
0.8%
0.1%

Figure S1 (2/3)

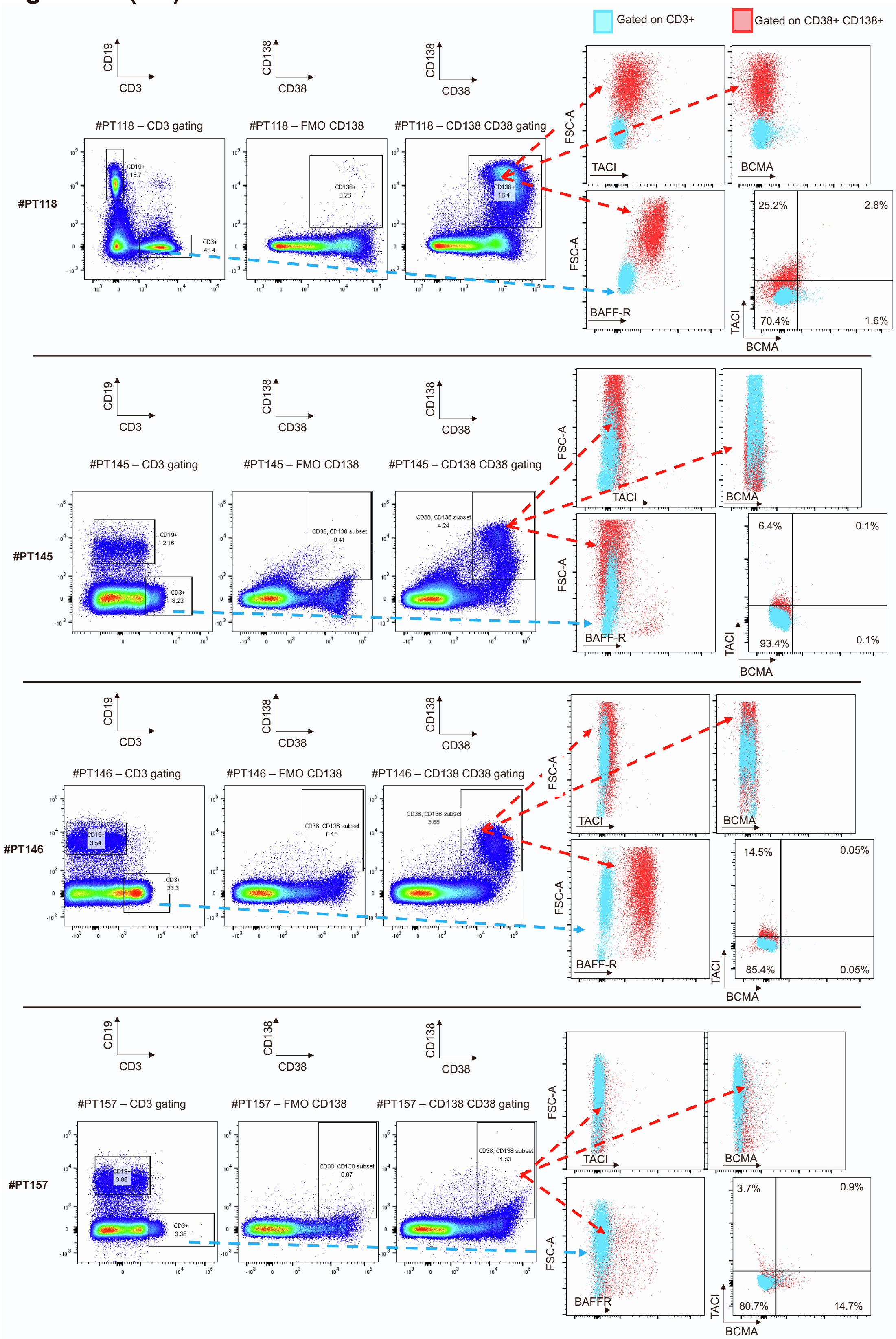

Gated on CD3+
Gated on CD38+ CD138+
#PT118
#PT118 – CD3 gating
#PT118 – FMO CD138
#PT118 – CD138 CD38 gating
CD19
CD3
CD138
CD38
FSC-A
TACI
BCMA
BAFF-R
25.2%
2.8%
70.4%
1.6%
#PT145
#PT145 – CD3 gating
#PT145 – FMO CD138
#PT145 – CD138 CD38 gating
6.4%
0.1%
93.4%
0.1%
#PT146
#PT146 – CD3 gating
#PT146 – FMO CD138
#PT146 – CD138 CD38 gating
14.5%
0.05%
85.4%
0.05%
#PT157
#PT157 – CD3 gating
#PT157 – FMO CD138
#PT157 – CD138 CD38 gating
BAFFR
3.7%
0.9%
80.7%
14.7%

Figure S1 (3/3)

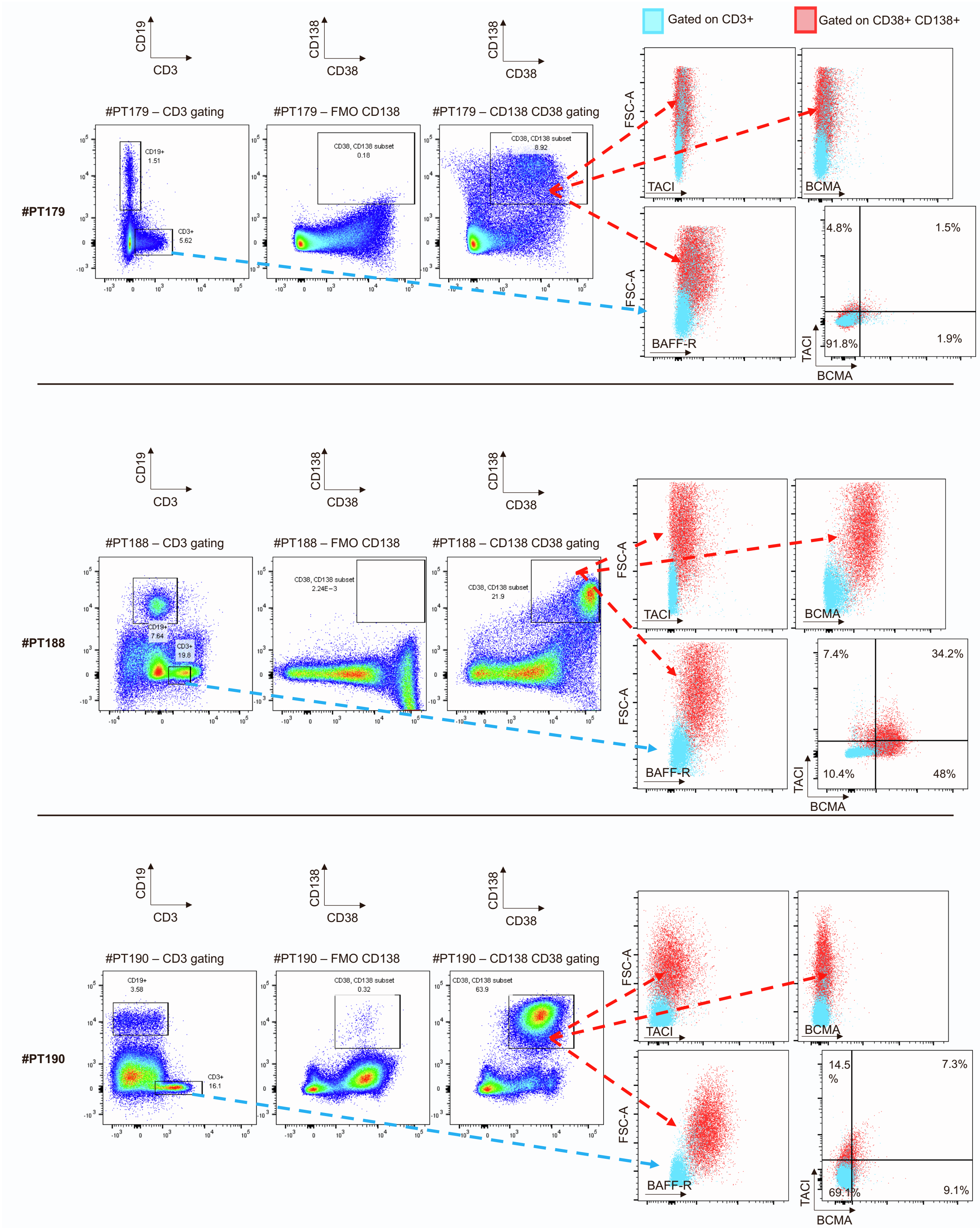

Gated on CD3+
Gated on CD38+ CD138+
#PT179
#PT179 – CD3 gating
#PT179 – FMO CD138
#PT179 – CD138 CD38 gating
CD19+ 1.51
CD3+ 5.62
CD38, CD138 subset 0.18
CD38, CD138 subset 8.92
CD19
CD3
CD138
CD38
FSC-A
TACI
BCMA
BAFF-R
4.8%
1.5%
91.8%
1.9%
#PT188
#PT188 – CD3 gating
#PT188 – FMO CD138
#PT188 – CD138 CD38 gating
CD19+ 7.64
CD3+ 19.8
CD38, CD138 subset 2.24E−3
CD38, CD138 subset 21.9
7.4%
34.2%
10.4%
48%
#PT190
#PT190 – CD3 gating
#PT190 – FMO CD138
#PT190 – CD138 CD38 gating
CD19+ 3.58
CD3+ 16.1
CD38, CD138 subset 0.32
CD38, CD138 subset 63.9
14.5 %
7.3%
69.1%
9.1%

Figure S1. Gating strategy used for flow cytometry analysis of BAFF-R, BCMA and TACI on malignant PCs.

(**A**) Cells were first gated on lymphocyte population in FSC-A / SSC-A, doublets were then excluded (FSC-A / FSC-H and SSC-A / SSC-H) and dead cells were excluded with DAPI live/dead staining. (**B**) Representative FCM plots for the gating of MM cells and the subsequent gating of target antigens are shown for n=10 patient samples. FCM plots show first on the left $CD3^+$ gating, used as negative control to determine BAFF-R, BCMA and TACI positive cells. Middle FCM plot shows fluorescence minus one (FMO) for CD138 used to correctly gate on $CD138^+$ MM cell population. FCM plot on the right shows patient sample stained with antibodies and the gating on $CD38^+CD138^+$ MM cells. FCM plots show BAFF-R, TACI, BCMA, and BCMA/TACI gating (red: MM population stained with antibodies) according to $CD3^+$ internal control (light blue).

Figure S2

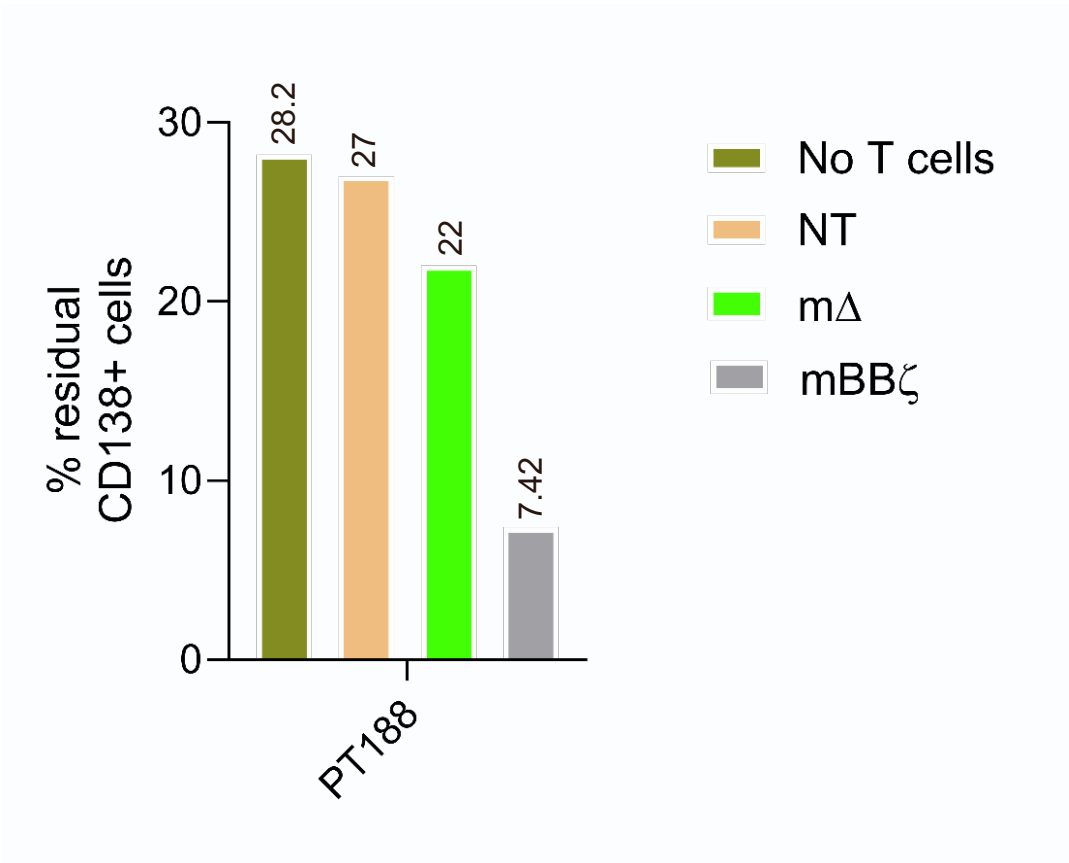

Figure S2. mBBζ MILs but not NT or mΔ MILs kill primary malignant PCs.

Residual CD138^{+} PCs after 24h of culture alone (No T cells), or co-culture with NT, mΔ or BBζ CAR-MILs.

Figure S3

A

Sample	Number of clonotypes
PT 118 NT	101426
PT 118 mΔ	186312
PT 118 BBζ	107041
PT 188 NT	110362
PT 188 mΔ	141565
PT 188 BBζ	123070
PT 190 NT	152099
PT 190 mΔ	49507
PT 190 BBζ	177901

B

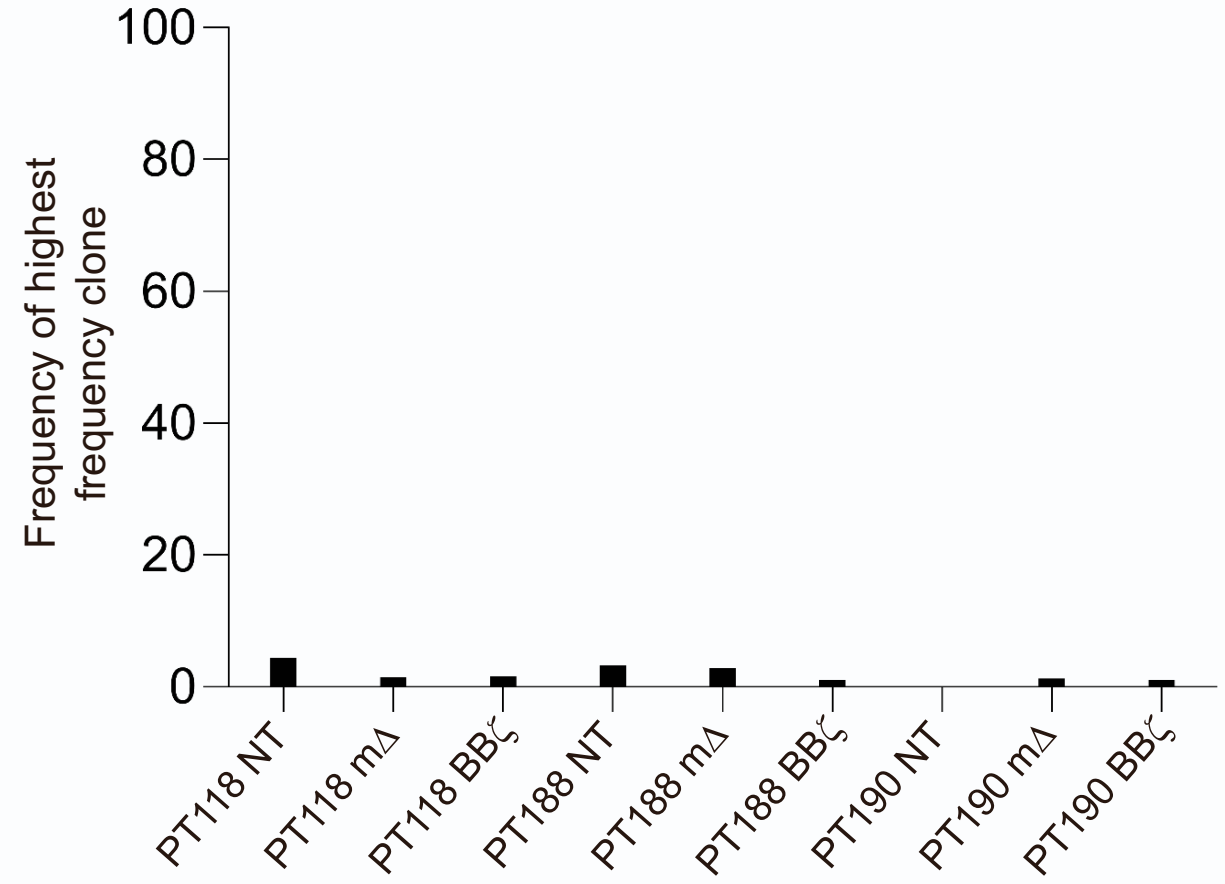

C

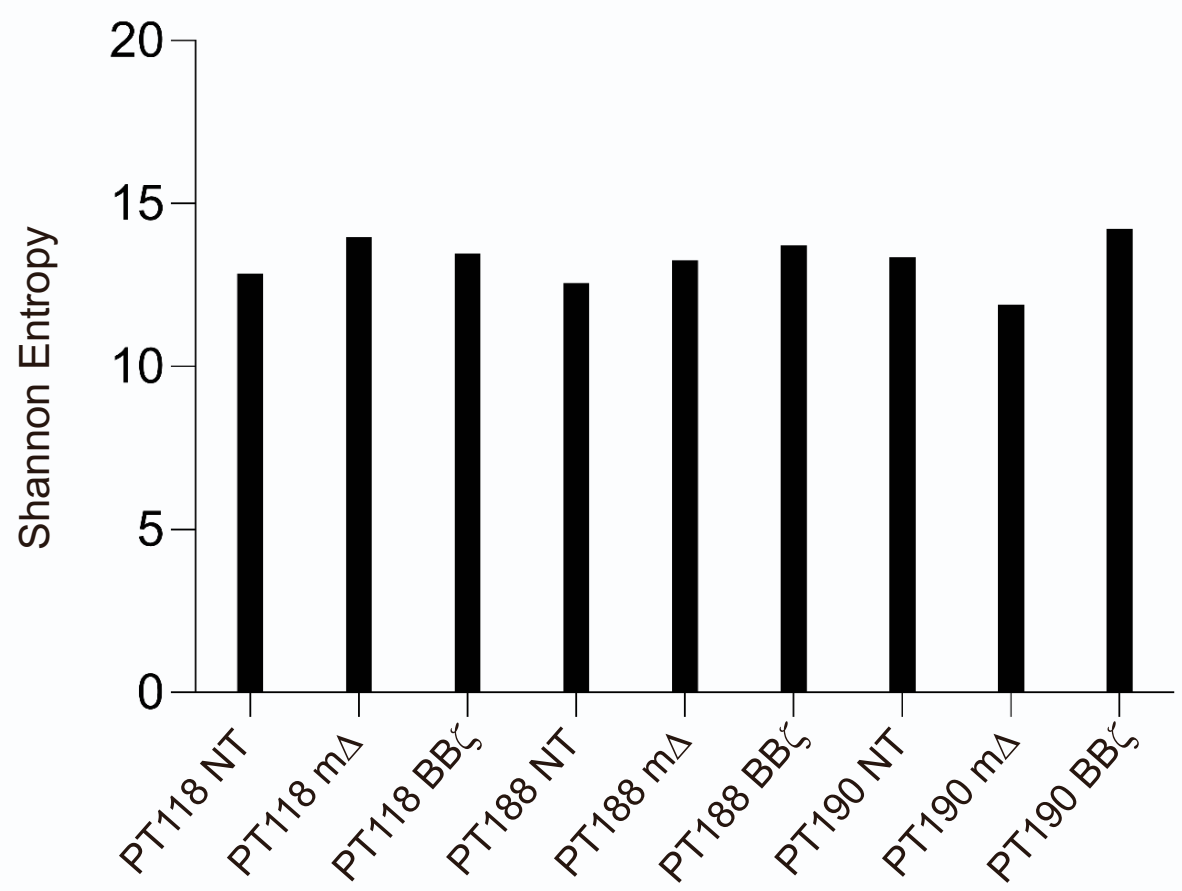

Figure S3. TCR β-chain sequencing results of MILs.

TCR β-chain sequencing was carried out for 3 patients (PT118, PT188, PT190). For each patient NT (non-transduced), mΔ and mBBζ MILs were analyzed. The number of clonotypes identified (**A**), the frequency of the most frequent clone (**B**) and the Shannon Entropy index (measure of TCR repertoire diversity) (**C**) are shown.

Figure S4

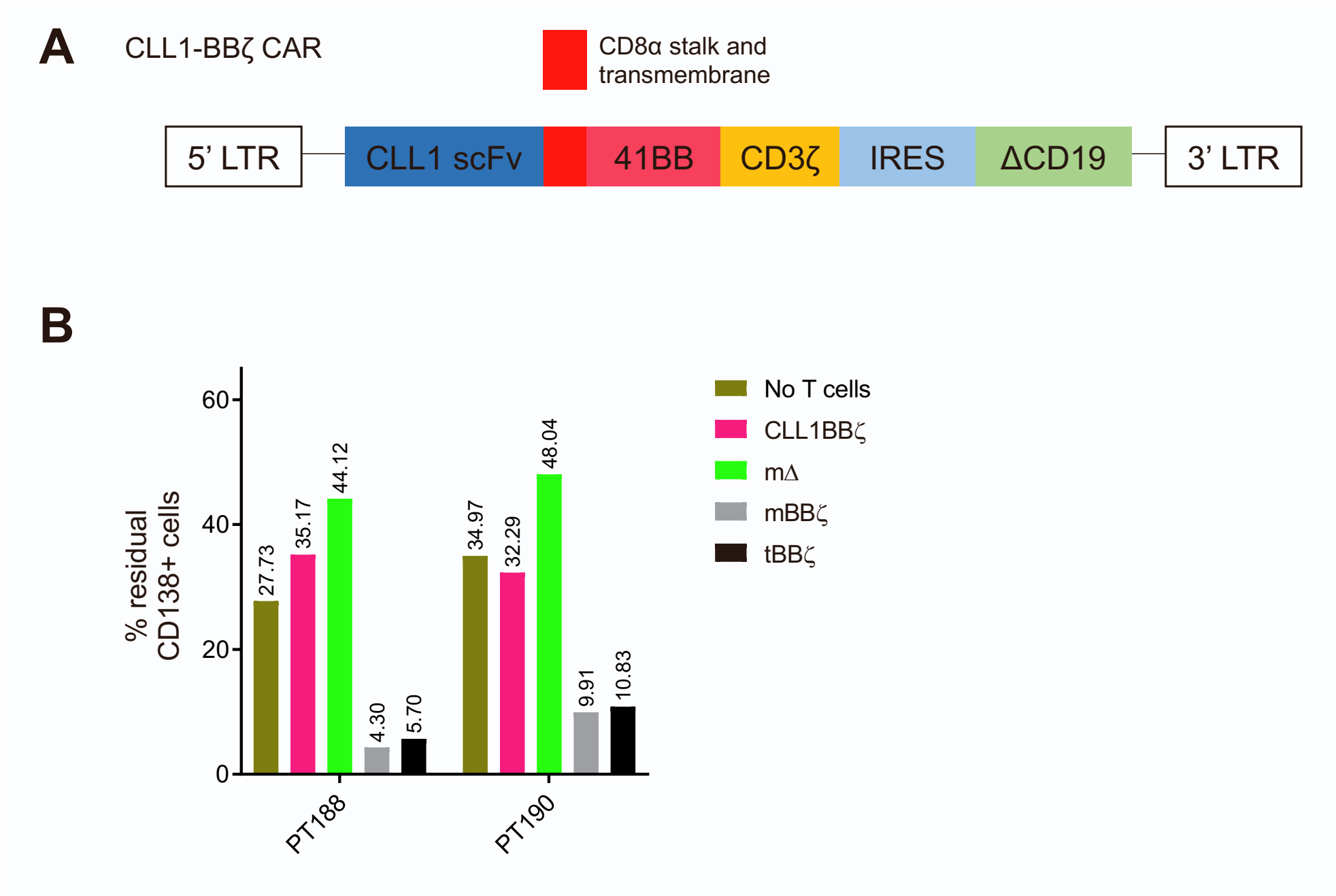

Figure S4. CLL1-BBζ CAR construct and CAR-MIL killing assay results.

(**A**) Scheme of CLL1-BBζ construct. (**B**) Primary malignant PCs derived from two MM patients were co-cultured with autologous APRIL or CLL1BBζ CAR-MILs for 24 hours and the % residual CD138+ malignant PCs was determined by FCM.

Figure S5 (1/5)

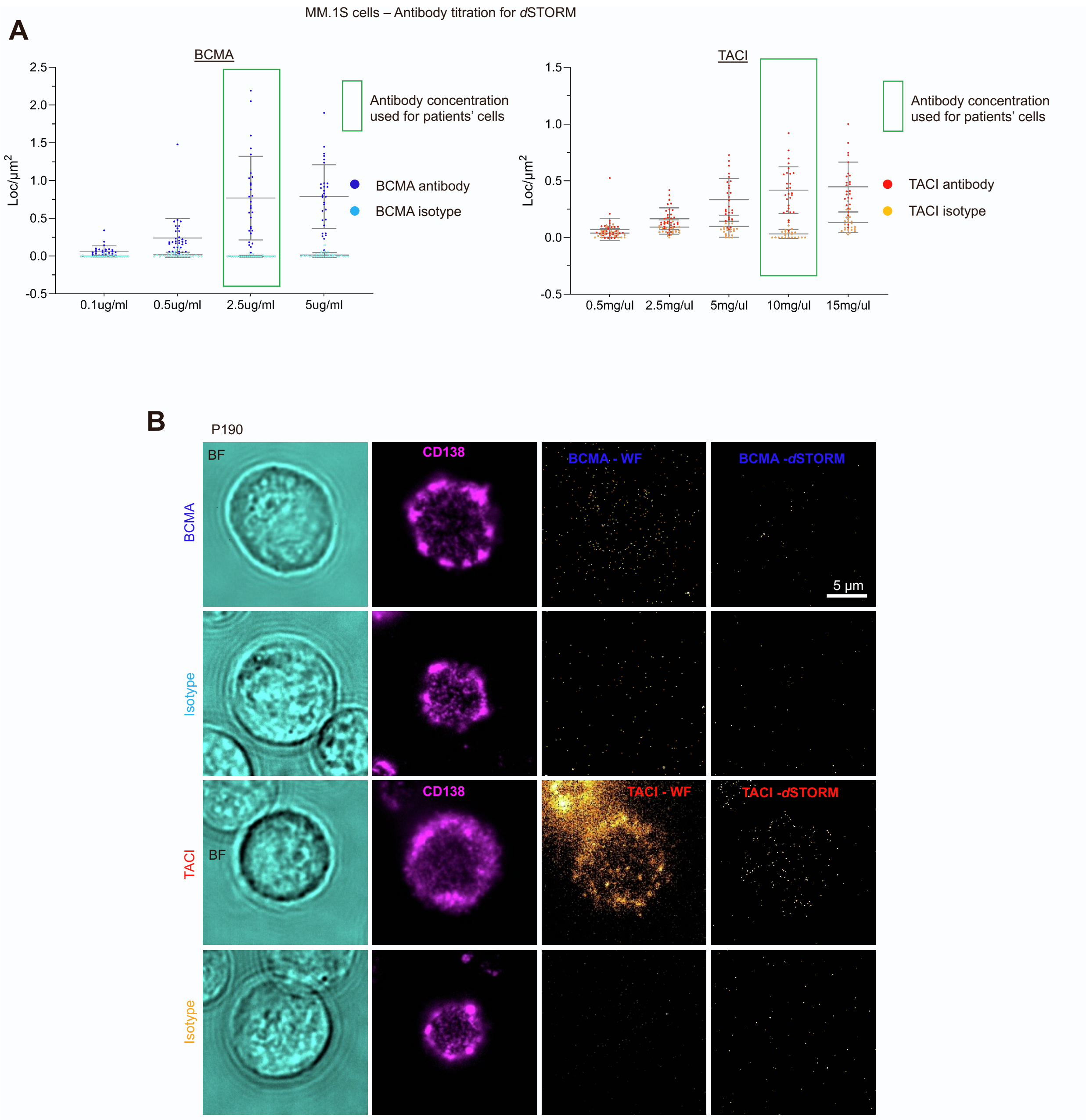
A
MM.1S cells – Antibody titration for dSTORM
BCMA
TACI
Loc/µm2
2.5
2.0
1.5
1.0
0.5
0.0
-0.5
0.1ug/ml
0.5ug/ml
2.5ug/ml
5ug/ml
Antibody concentration used for patients' cells
BCMA antibody
BCMA isotype
0.5mg/ul
2.5mg/ul
5mg/ul
10mg/ul
15mg/ul
TACI antibody
TACI isotype
B
P190
BF
CD138
BCMA - WF
BCMA -dSTORM
5 µm
BCMA
Isotype
TACI
TACI - WF
TACI -dSTORM
Isotype

Figure S5 (2/5)

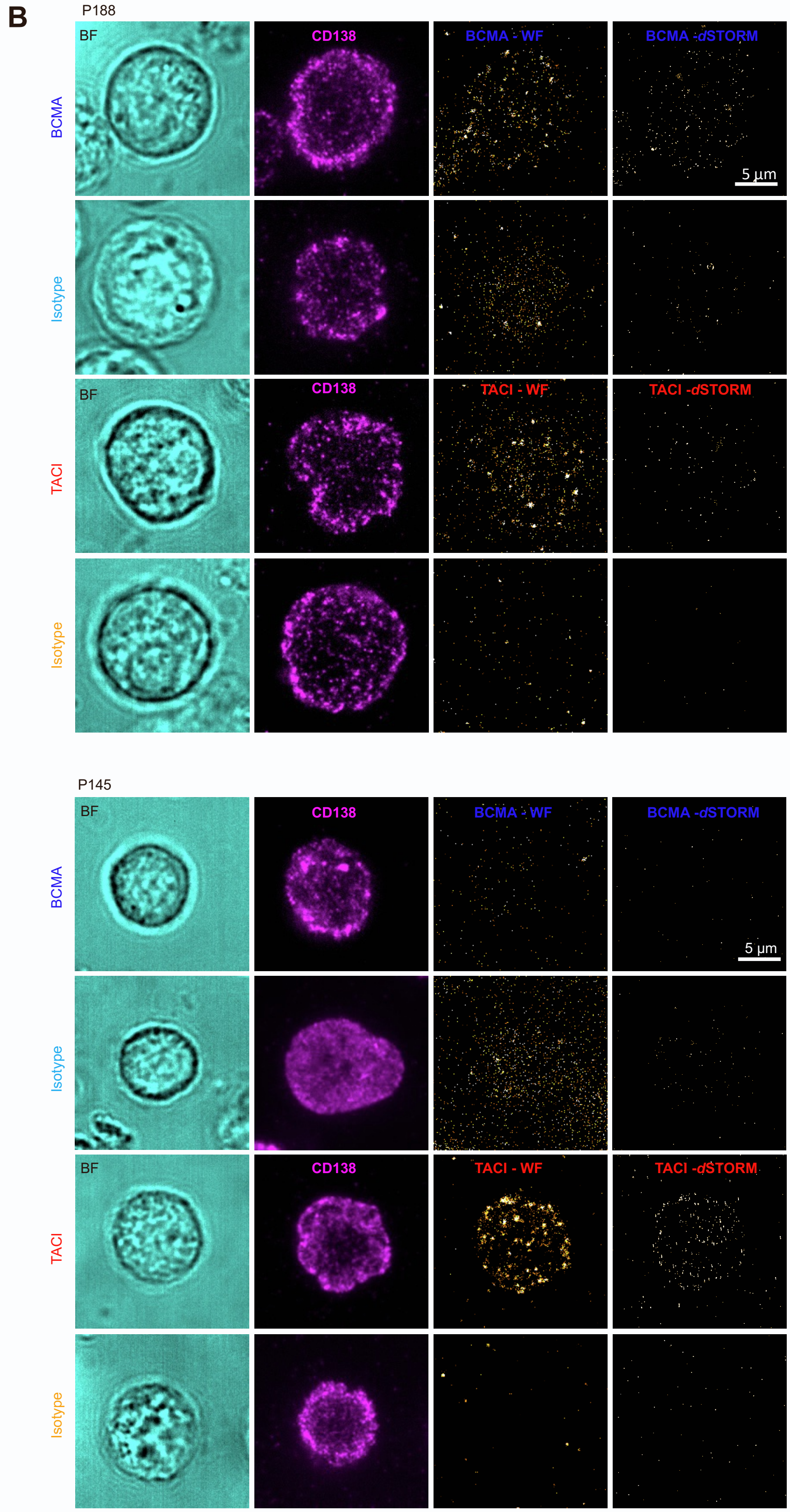

B
P188
BF
CD138
BCMA - WF
BCMA -dSTORM
BCMA
5 µm
Isotype
BF
CD138
TACI - WF
TACI -dSTORM
TACI
Isotype
P145
BF
CD138
BCMA - WF
BCMA -dSTORM
BCMA
5 µm
Isotype
BF
CD138
TACI - WF
TACI -dSTORM
TACI
Isotype

Figure S5 (3/5)

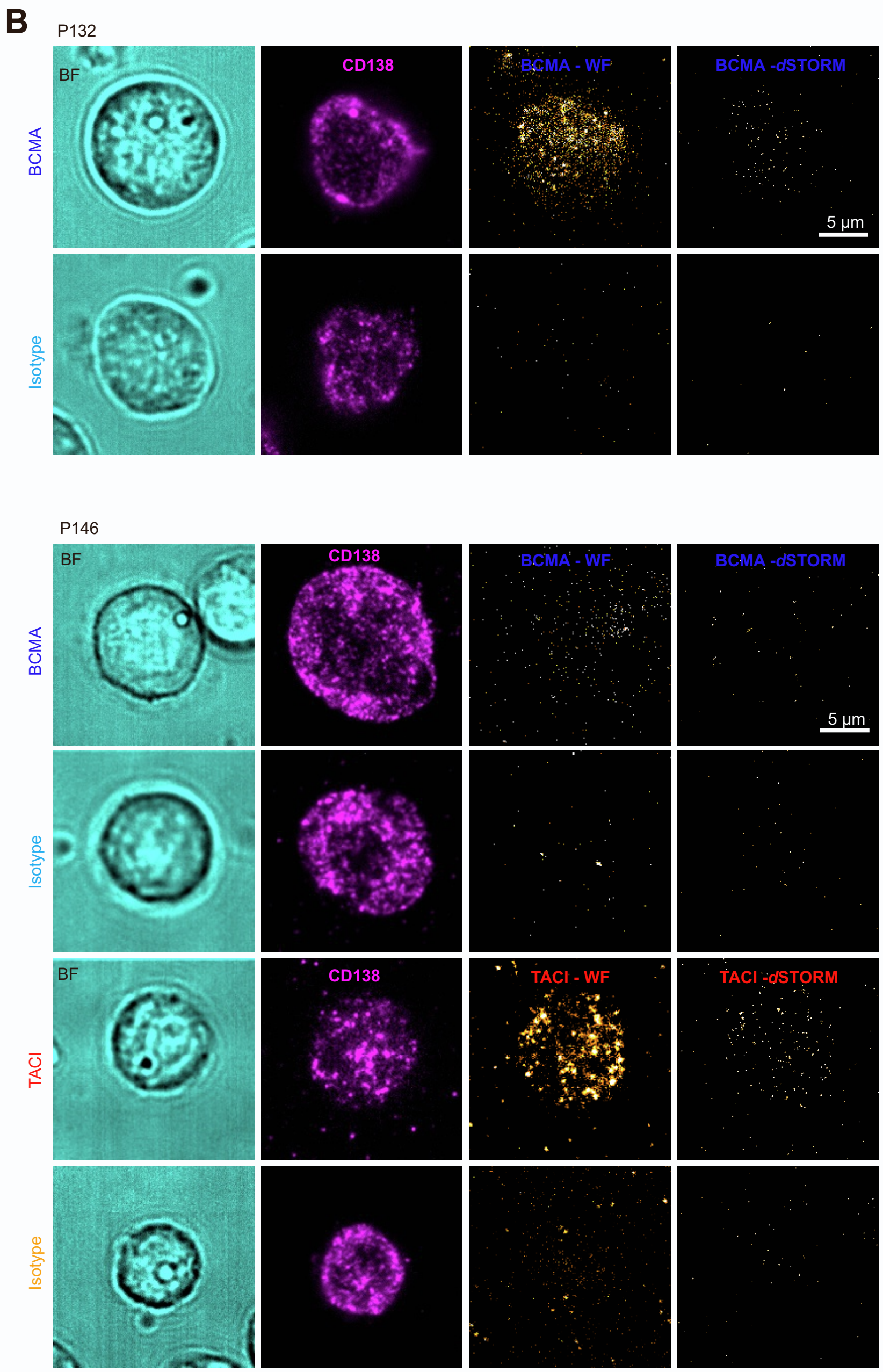

B
P132
BF
BCMA
CD138
BCMA - WF
BCMA -dSTORM
5 µm
Isotype
P146
BF
BCMA
CD138
BCMA - WF
BCMA -dSTORM
5 µm
Isotype
BF
TACI
CD138
TACI - WF
TACI -dSTORM
Isotype

Figure S5 (4/5)

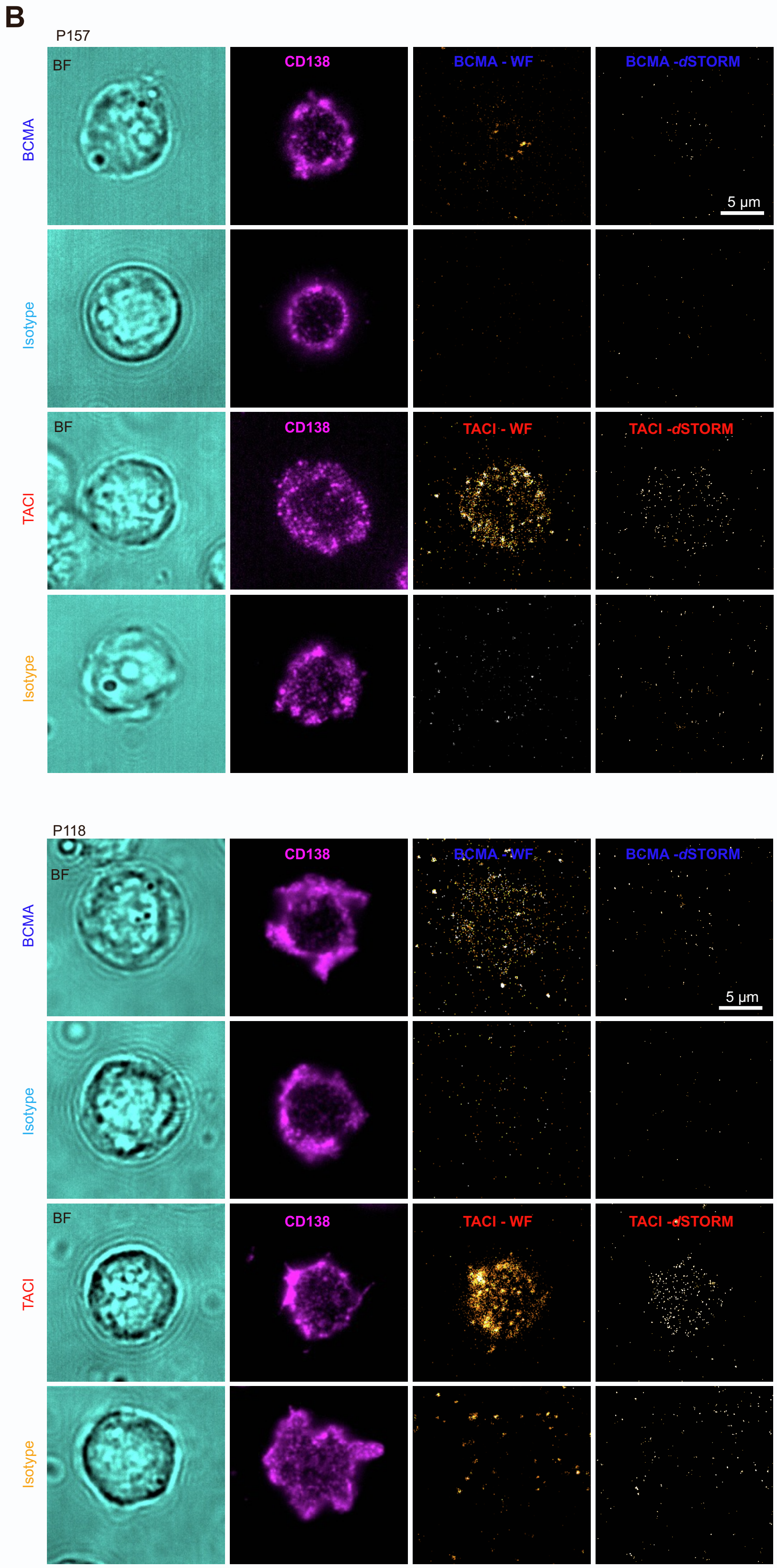
B
P157
BF
CD138
BCMA - WF
BCMA -dSTORM
BCMA
5 µm
Isotype
BF
CD138
TACI - WF
TACI -dSTORM
TACI
Isotype
P118
BF
CD138
BCMA - WF
BCMA -dSTORM
BCMA
5 µm
Isotype
BF
CD138
TACI - WF
TACI -dSTORM
TACI
Isotype

Figure S5 (5/5)

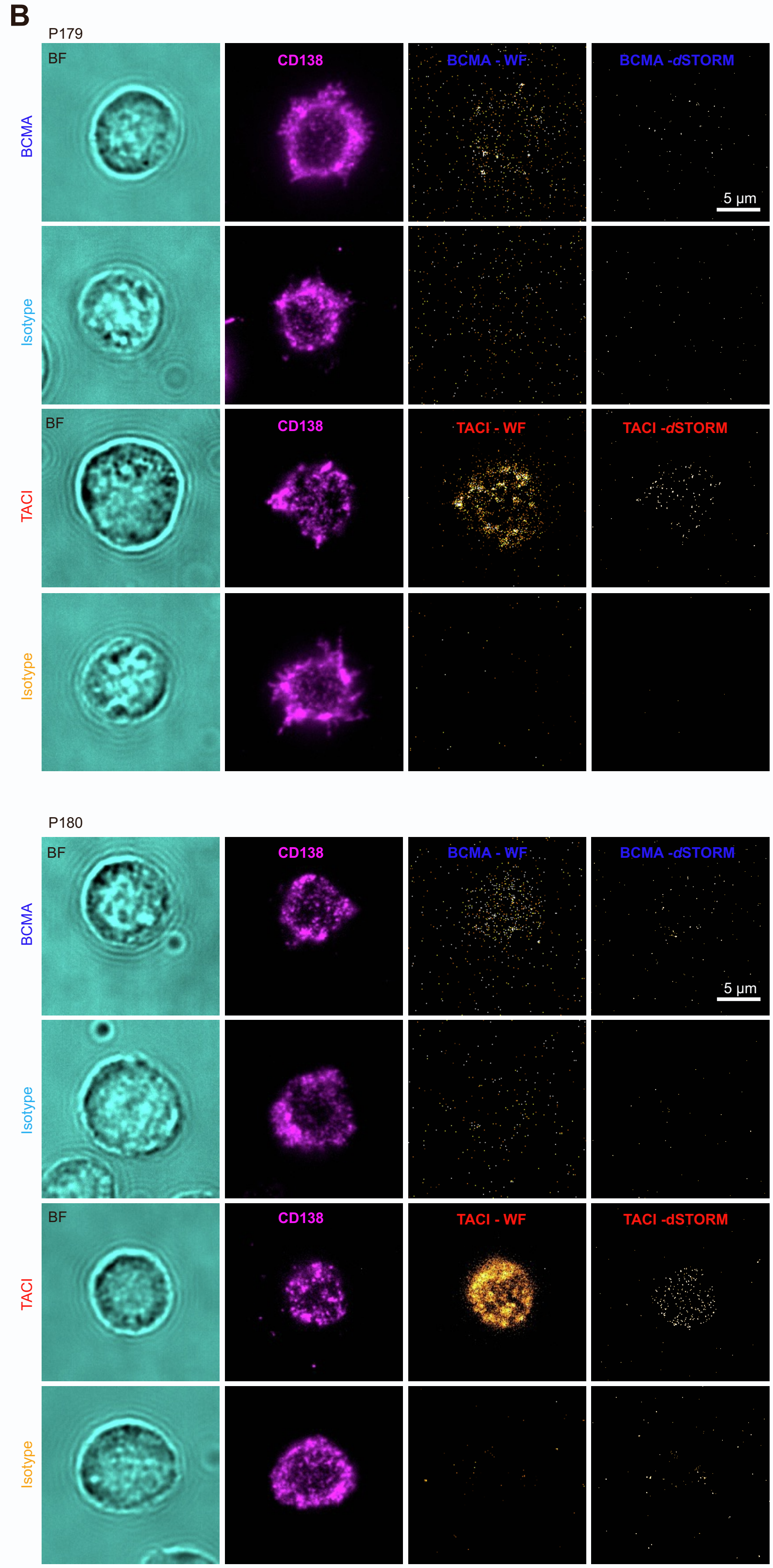
B
P179
BF
CD138
BCMA - WF
BCMA -dSTORM
BCMA
5 µm
Isotype
BF
CD138
TACI - WF
TACI -dSTORM
TACI
Isotype
P180
BF
CD138
BCMA - WF
BCMA -dSTORM
BCMA
5 µm
Isotype
BF
CD138
TACI - WF
TACI -dSTORM
TACI
Isotype

Figure S5. Titration of BCMA and TACI antibodies for *d*STORM and representative super-resolution microscopy images of primary malignant PCs.

(**A**) Titration of anti-BCMA (left) and anti-TACI (right) antibodies compared to matched isotype controls, on MM.1S cells expressing both target antigens. (**B**) Representative *d*STORM microscopy images for each patient sample analysed. From left to right: BF, bright field image; CD138 staining; WF, wide field image and *d*STORM super-resolution reconstructed image.

Figure S6

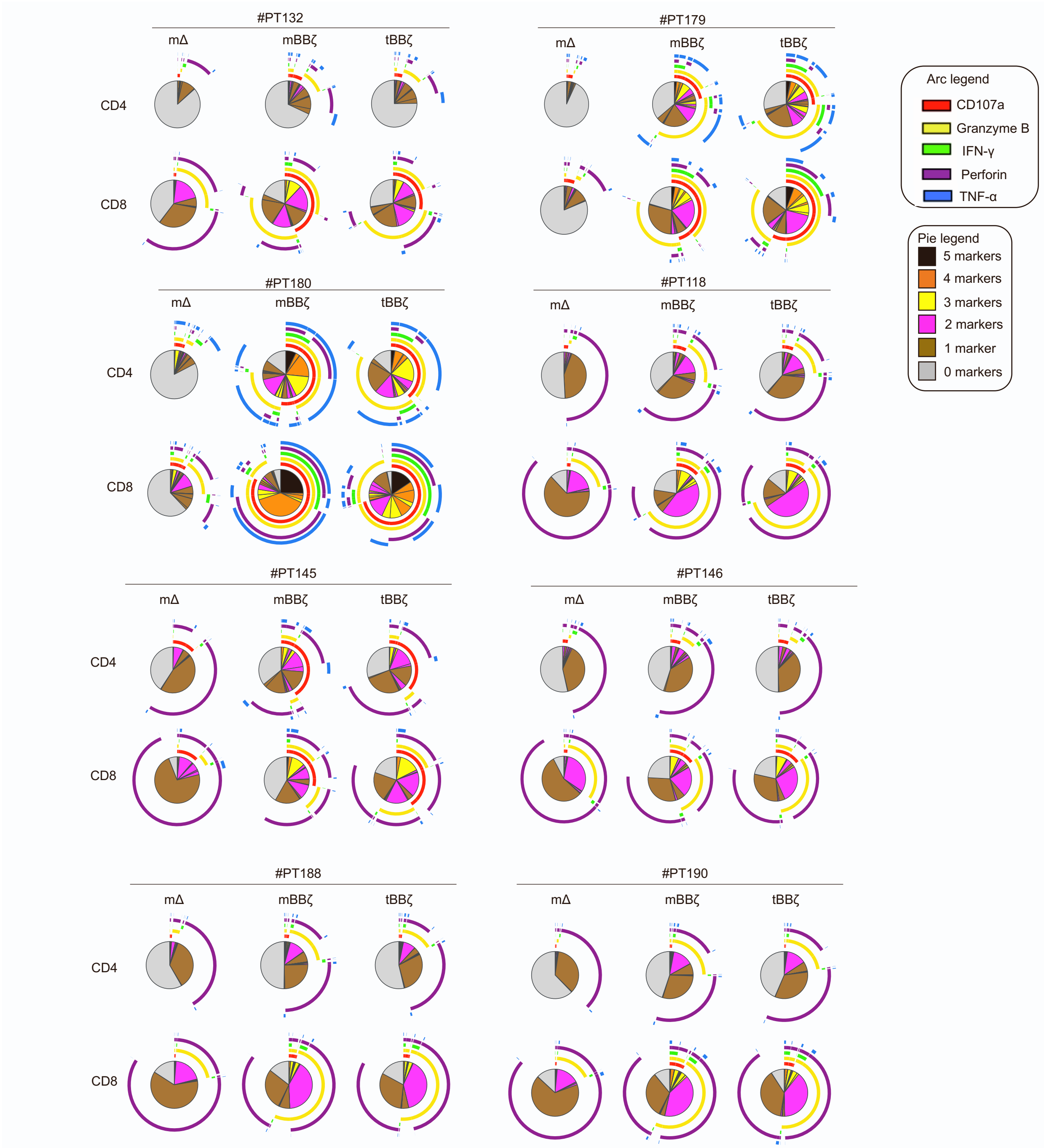

#PT132
mΔ
mBBζ
tBBζ
CD4
CD8
#PT179
mΔ
mBBζ
tBBζ
#PT180
mΔ
mBBζ
tBBζ
CD4
CD8
#PT118
mΔ
mBBζ
tBBζ
#PT145
mΔ
mBBζ
tBBζ
CD4
CD8
#PT146
mΔ
mBBζ
tBBζ
#PT188
mΔ
mBBζ
tBBζ
CD4
CD8
#PT190
mΔ
mBBζ
tBBζ
Arc legend
CD107a
Granzyme B
IFN-γ
Perforin
TNF-α
Pie legend
5 markers
4 markers
3 markers
2 markers
1 marker
0 markers

Figure S6. Intracellular cytokine staining and polyfunctionality of CAR-MILs after co-culture with autologous PCs.

CAR-MILs were co-cultured with autologous primary malignant PCs for 6 hours and then analysed by ICS. SPICE plots depict polyfunctionality of control mΔ compared to mBBζ or tBBζ CAR-MILs. The pies depict all different combinations of populations expressing 5, 4, 3, 2, 1 or 0 markers. The arcs around the pie indicate which cytokines were detected.

Figure S7

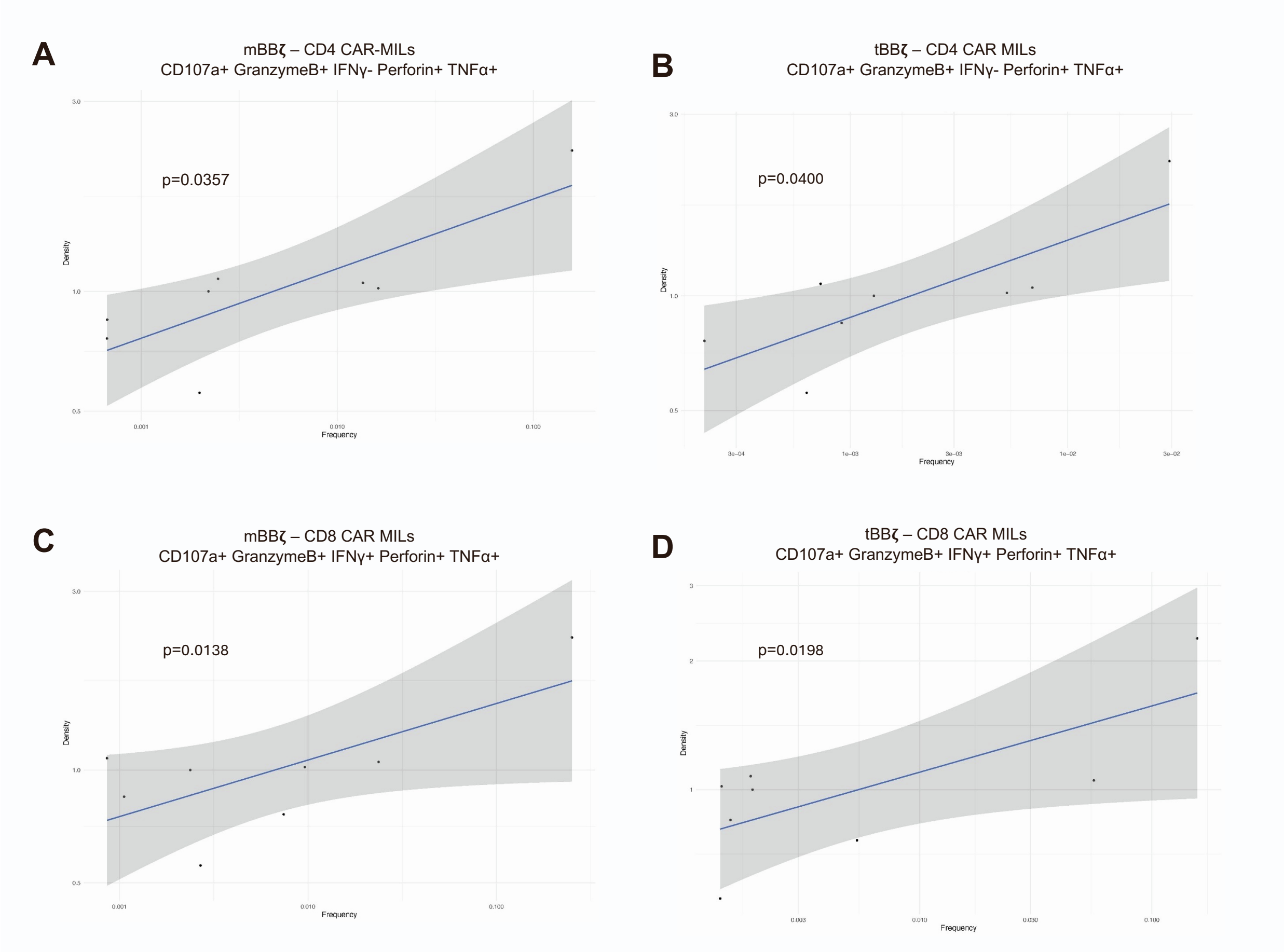

Figure S7. Correlation between combined TACI and BCMA antigen densities and CAR-MIL polyfunctionality.

Spearman correlations between CAR-MIL polyfunctionality and combined BCMA and TACI antigen density show that higher BCMA/TACI antigen densities associate with higher CD4 (**A-B**) and CD8 (**C-D**) CAR-MIL polyfunctionality for both 4 markers (**A, C**) or 5 markers (**B, D**). Statistical analyses for the spearman correlation coefficients and associated p-values were calculated with the cor function in R.

Figure S8 (1/2)

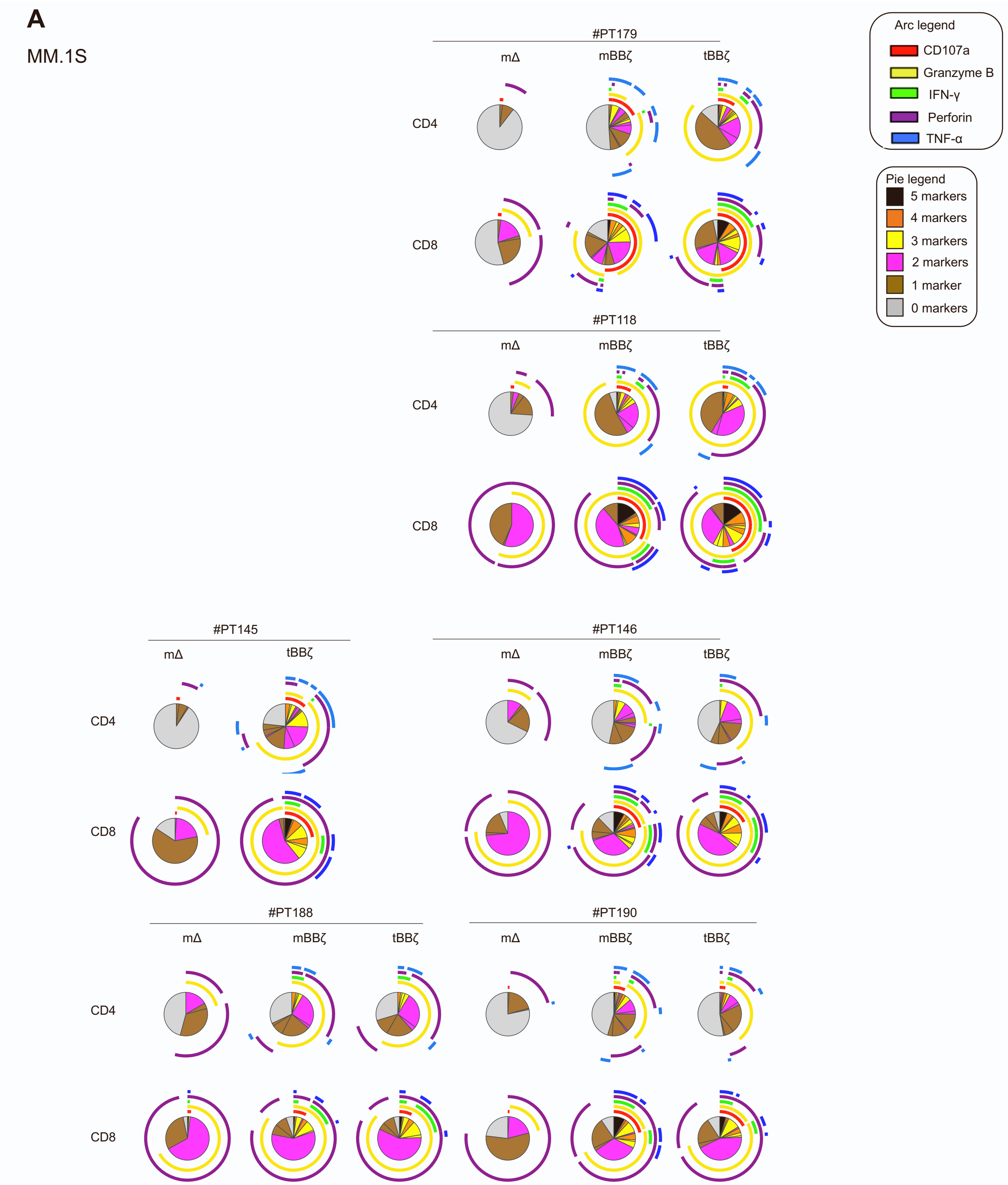

A
MM.1S
#PT179
mΔ
mBBζ
tBBζ
CD4
CD8
#PT118
mΔ
mBBζ
tBBζ
CD4
CD8
#PT145
mΔ
tBBζ
CD4
CD8
#PT146
mΔ
mBBζ
tBBζ
#PT188
mΔ
mBBζ
tBBζ
CD4
CD8
#PT190
mΔ
mBBζ
tBBζ
Arc legend
CD107a
Granzyme B
IFN-γ
Perforin
TNF-α
Pie legend
5 markers
4 markers
3 markers
2 markers
1 marker
0 markers

Figure S8 (2/2)

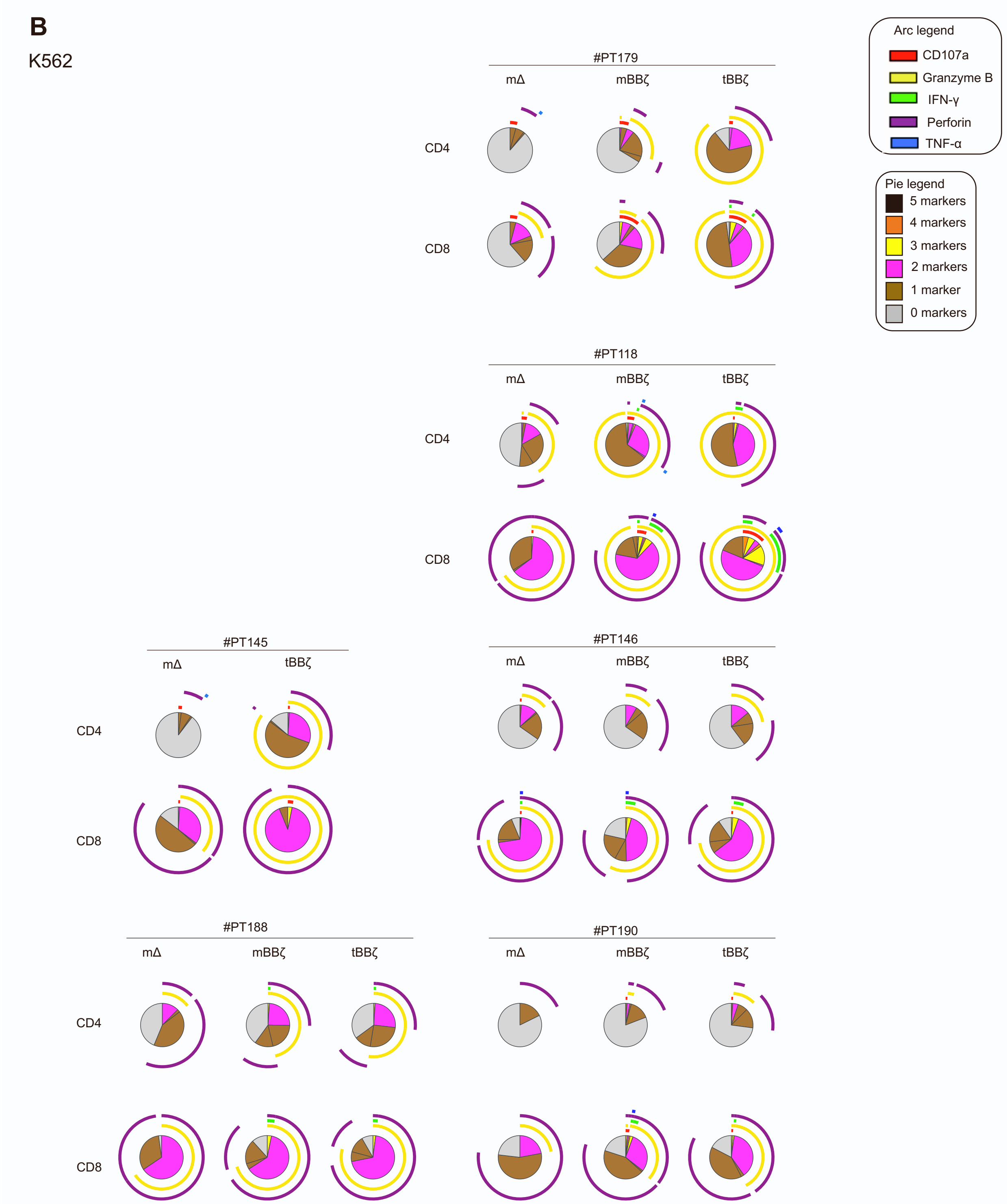
B
K562
#PT179
mΔ
mBBζ
tBBζ
CD4
CD8
#PT118
mΔ
mBBζ
tBBζ
CD4
CD8
#PT145
mΔ
tBBζ
CD4
CD8
#PT146
mΔ
mBBζ
tBBζ
#PT188
mΔ
mBBζ
tBBζ
CD4
CD8
#PT190
mΔ
mBBζ
tBBζ
Arc legend
CD107a
Granzyme B
IFN-γ
Perforin
TNF-α
Pie legend
5 markers
4 markers
3 markers
2 markers
1 marker
0 markers

Figure S8. Intracellular cytokine staining and polyfunctionality of CAR-MILs after co-culture with cell lines.

CAR-MILs were co-cultured with the $BCMA^{+}TACI^{+}$ cell line MM.1S (**A**) or with the $BCMA^{-}$ $TACI^{-}$ cell line K562 (**B**), for 6 hours, and then analysed by ICS. SPICE plots depict polyfunctionality of control m∆ compared to mBBζ or tBBζ CAR-MILs. The pies depict all different combinations of populations expressing 5, 4, 3, 2, 1 or 0 markers. The arcs around the pie indicate which cytokines were detected.
